# Characterization of an engineered live bacterial therapeutic for the treatment of phenylketonuria in a human gut-on-a-chip

**DOI:** 10.1038/s41467-021-23072-5

**Published:** 2021-05-14

**Authors:** M. Tyler Nelson, Mark R. Charbonneau, Heidi G. Coia, Mary J. Castillo, Corey Holt, Eric S. Greenwood, Peter J. Robinson, Elaine A. Merrill, David Lubkowicz, Camilla A. Mauzy

**Affiliations:** 1 United States Air Force Research Laboratory, 711th Human Performance Wing, Airman Systems Directorate, Bioengineering Division, Wright-Patterson AFB, OH Columbus, USA; 2 Synlogic Inc, Cambridge, MA USA; 3 National Research Council, The National Academies of Sciences, Engineering, and Medicine, Washington DC, USA; 4 Oak Ridge Institute for Science and Education, Oak Ridge, TN USA; 5 The Henry M. Jackson Foundation, Bethesda, MD USA

**Keywords:** Metabolic engineering, Synthetic biology, Phenotypic screening

## Abstract

Engineered bacteria (synthetic biotics) represent a new class of therapeutics that leverage the tools of synthetic biology. Translational testing strategies are required to predict synthetic biotic function in the human body. Gut-on-a-chip microfluidics technology presents an opportunity to characterize strain function within a simulated human gastrointestinal tract. Here, we apply a human gut-chip model and a synthetic biotic designed for the treatment of phenylketonuria to demonstrate dose-dependent production of a strain-specific biomarker, to describe human tissue responses to the engineered strain, and to show reduced blood phenylalanine accumulation after administration of the engineered strain. Lastly, we show how in vitro gut-chip models can be used to construct mechanistic models of strain activity and recapitulate the behavior of the engineered strain in a non-human primate model. These data demonstrate that gut-chip models, together with mechanistic models, provide a framework to predict the function of candidate strains in vivo.

## Abstract (web summary)

Engineered live bacteria could represent a new class of therapeutic treatment for human disease. Here, the authors use a human gut-on-a-chip microfluidics system to characterize an engineered live bacterial therapeutic, designed for the treatment of phenylketonuria, and to construct mathematical models that predict therapeutic strain function in non-human primates.

## Introduction

Phenylketonuria (PKU) is a rare genetic disease that results in reduced activity of the enzyme, phenylalanine hydroxylase, which converts the essential amino acid, phenylalanine (Phe) to tyrosine. For patients with PKU, dietary protein consumption causes prolonged elevation of plasma Phe concentrations and can lead to severe cognitive impairment, among other sequelae^1,2^. There is increasing evidence that live biotherapeutics comprised of engineered microbes (synthetic biotics) can be used to sense and respond to environmental signals within the human body in order to metabolize potentially toxic compounds, including Phe^3^. We recently described the development of a synthetic biotic strain of *E. coli* Nissle 1917, called SYNB1618, designed to consume Phe in the human upper gastrointestinal tract^4^. SYNB1618 degrades Phe by the expression of two distinct mechanisms: (1) the conversion of Phe to *trans*-cinnamic acid (TCA) by phenylalanine ammonia lyase (PAL), and (2) the conversion of Phe to phenylpyruvic acid by l-amino acid deaminase (LAAD). TCA is further converted to hippuric acid (HA) in vivo and excreted in urine. We have previously shown that oral administration of SYNB1618 significantly lowered blood Phe concentrations in a mouse model of PKU and resulted in dose-dependent production of the PAL-specific urinary biomarker, HA, in healthy non-human primates (NHP). A Phase 1/2a dose escalation study in healthy volunteers and PKU patients demonstrated that SYNB1618 was generally well tolerated (Clinicaltrials.gov Identifier: NCT03516487). This study also revealed a dose-dependent production of urinary HA upon administration of SYNB1618, confirming Phe consumption by the synthetic biotic in human subjects.

The tools of synthetic biology enable rapid and cost-effective development of engineered strain prototypes^5^. However, clinical development of therapeutics requires compliance with strict regulatory guidelines and does not scale similarly^3^. Therefore, it is essential to develop testing strategies that can be applied early in the development process to characterize the function of synthetic biotics, to optimize potency, and to establish confidence in their translational potential. For engineered bacterial therapeutics, environmental conditions are important determinants of strain viability and metabolism. Methods that simulate the conditions of the human upper gastrointestinal tract, such as the simulated human intestinal microbial ecosystem (SHIME), are useful for characterizing the viability and function of engineered strains^6^. However, these simplified in vitro simulations lack host cells and tissue architecture. By contrast, animal models may be used to study synthetic biotic function in the context of the complete host organism, though the translational value of animal models varies by species and genotype, due in part to differences in gastrointestinal (GI) physiology with respect to humans^7^.

In recent years, significant advances have been made toward organ-on-a-chip (OOC) microfluidic systems that can be used to study the effects of engineered microbes and their products on human tissues, including effects on tissue viability^8^. These microscale synthetic tissue surrogates enable robust cellular and molecular analysis, fine control over transport and fluid flow dynamics, and incorporation of complex mechanical stimuli that capture aspects of human gut physiology. Gut-on-a-chip in vitro models have shown promise in overcoming many limitations of conventional cell culture techniques (e.g., Transwells^®^) that fail to recapitulate the complexity and function of human gut tissue^9,10^. For example, dynamic culturing conditions in microfluidic systems enhance gut-endothelial barrier health, aid in maintaining microbiome homeostasis, and facilitate nutrient and metabolite transport under steady-state conditions. Microfluidic cultures that incorporate both passive and applied mechanical forces can also drive the development of human gut-like villus projections as well as the formation of a functional mucus layer^11–13^. The benefits of dynamic culture are not limited to host responses but also address significant experimental obstacles. In particular, dynamic systems can suppress bacterial overgrowth of the host cell culture and limit inflammatory responses^11,13^. This technology represents an opportunity to simulate synthetic biotic activity in the human body, as well as bacterial clearance and the flux of bacterial and host metabolites between body compartments in a dynamic environment (e.g., gut lumen and blood). Moreover, mechanistic data from OOC systems can be used to calibrate predictive mechanistic models of in vivo strain activity.

In this article, we aim to determine whether a human gut-chip model, representing the upper gastrointestinal tract, could recapitulate the in vivo activity of a synthetic biotic designed for the treatment of PKU. Single bolus application of a synthetic live biotherapeutic, SYN5183, is applied to the gut-compartment resulting in dose dependent increases in the biomarker, trans-cinnamic acid (TCA), and a corresponding 26.9% decrease in systemic Phe. Simulations performed using a mathematical model, calibrated to in vitro gut-chip results, showed a high degree of correlation with previously published non-human primate results^4^, highlighting the predictive potential of gut-chip technology to accelerate synbiotic development.

## Results

### Gut-chip model characterization

**Fig. 1:**
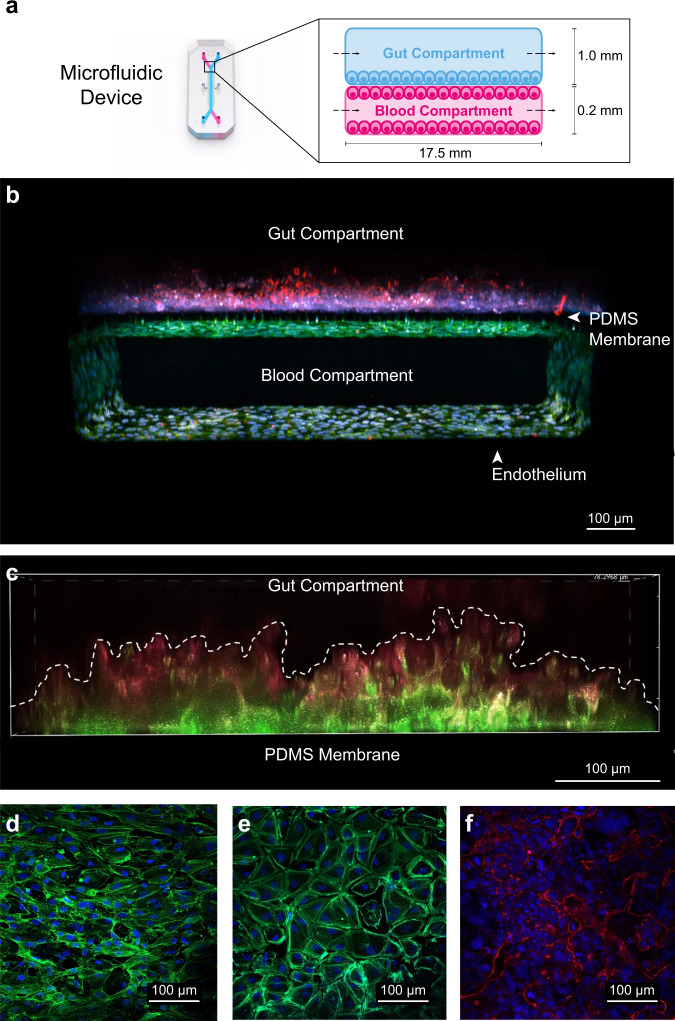
Characterization of a microfluidic human gut-chip model. **a** Schematic representation of two-compartment gut-chip model design. Human enterocyte-like (Caco2-BBE) and goblet-like cells (HT-29 MTX) were co-cultured in the upper channel (gut compartment) and interfaced with microvascular endothelial cells in the lower channel (blood compartment), through a thin, porous, flexible membrane. (**b**, *n* = 3 gut-chips) Confocal z-stack micrograph of the human gut-chip model, displaying the fully vascularized endothelial channel (bottom, green—ZO-1) and its interface with the gut epithelial barrier (upper channel, red—ZO-1) post-10 days of maturation. (**c**, *n* = 3 gut chips) Continuous dynamic culture (stretch 10% strain, 0.25 Hz and physiological flow = 60 µL/h; 0.0003 dynes/cm^2^) of the Caco2 intestinal cells leads to spontaneous 3D morphogenesis, producing villus-like projections (120–150 µm, red—phalloidin Alexa-fluor 555, green—ZO-1) within the gut compartment. (**d**–**f**, *n* = 2 gut chips) Micrographs of the crucial gut-vascular interface displaying endothelial tight junctions (**d**, **e**; green—ZO-1, blue—DAPI) and gut epithelial tight junctions (**f**; red—ZO-1, blue—DAPI).

A human gut-chip model was developed to simulate the metabolic activity of a synthetic biotic in the human gastrointestinal tract. Fig. 1a displays the two-compartment microfluidic device^11^ and a schematic describing the orientation of gut and endothelial cell components. Human enterocyte-like (Caco2-BBE) and goblet-like cells (HT-29 MTX) were cultured in the upper channel and interfaced with microvascular endothelial cells (immortalized cell line) through a thin, porous, flexible membrane, creating a gut-blood barrier. Fig. 1b displays a confocal z-stack image of the gut-chip, highlighting the fully vascularized channel and the direct interface with the gut epithelial surface, where cellular projections extend through the pores, resulting in physical association. Void channels running parallel to and offset from the fluidic channels provide vacuum-induced suction, creating cyclical stretch forces on the membrane simulating peristaltic strain. Continuous dynamic culture (stretch and physiological flow = 60 µL/h; 0.0003 dynes/cm^2^) of the Caco2 intestinal cells leads to spontaneous 3D morphogenesis, producing villus-like projections (120–150 µm) within the gut compartment (Fig. 1c). Immunofluorescent staining of f-actin and ZO-1 (tight junctions) in the gut epithelial cells show that the finger-like projections extend well beyond the membrane surface and maintain consistent morphologies and staining throughout the length of the channel. Targeted imaging of endothelial cells at the membrane (Fig. 1d) or on the bottom surface of the lower channel (Fig. 1e) reveals a continuous, honeycomb patterned ZO-1 tight-junctional protein staining. Fig. 1f shows a high magnification image of gut epithelial cell tight-junction formation (ZO-1, Red). These observations suggest that the human gut-chip exhibits several physiological features and molecular signatures of a functional gut-blood barrier and that this model system is suitable for evaluating synthetic biotic function and biomarker transport.

### Activity of SYN5183 in a human gut-chip

**Fig. 2:**
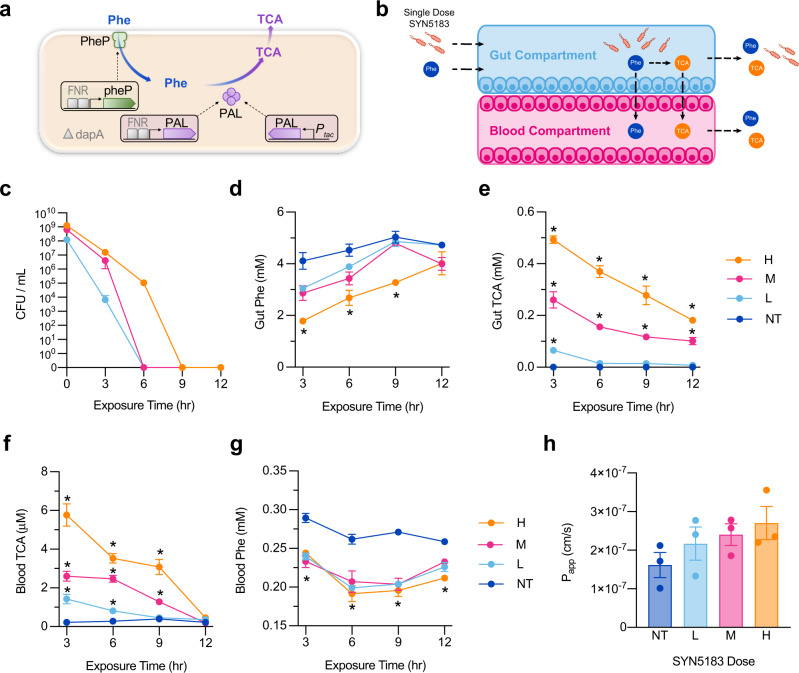
Administration of single dose SYN5183 in a human gut-chip model. **a** Schematic representation of SYN5183 design. SYN5183 contains chromosomally inserted genes encoding PheP, a high-affinity Phe transporter that can bring Phe into the cytoplasm, and PAL, which converts Phe to TCA. Induction of these components is carried out partially by the anaerobic-responsive transcriptional activator FNR (fumarate and nitrate reductase regulator), for activation of PAL and PheP. An additional copy of PAL is placed under the control of an Isopropyl β-D-1-thiogalactopyranoside (IPTG) inducible promoter, P_*tac*_, for strain activation during production of drug product. Δ*dapA* indicates deletion of the *dapA* gene, leading to diaminopimelic acid auxotrophy. **b** Schematic of study design. SYN5183 was administered as a single bolus dose at the start of the experiment in the gut compartment, and SIF containing 5.0 mM Phe was continuously administered to the gut compartment. SYN5183 CFU were enumerated from gut compartment effluents, and Phe and TCA were quantified using effluents collected from the gut and blood compartments. **c**–**e** Concentrations of SYN5183 (**c**; CFU/mL, *n* = 3 chips), Phe (**d**; mM, *n* = 3 chips), and TCA (**e**; mM, *n* = 3 chips) recovered from gut compartment effluents over time post-dose. **f**, **g** Concentrations of TCA (**f**; mM, *n* = 3 chips) and Phe (**g**; mM, *n* = 3 chips) recovered from blood compartment effluents over time post-dose. **h** Apparent permeability (*P*_app_; cm/s) across the gut barrier 12 h post-dose. For (**c**–**h**), H, M, and L correspond to SYN5183 doses of 1.25 × 10^9^ CFU/mL, 6.25 × 10^8^ CFU/mL, and 1.25 × 10^8^ CFU/mL, respectively. NT corresponds to non-treated chips. *p < 0.05, 2-way ANOVA, with a post-hoc Tukey analysis using a 95% confidence interval compared to the NT group, *n* = 6 independent gut chips, error bars represent SEM with the middle point representing the mean value. Source data are provided as a Source Data file.

Activity of the LAAD enzyme, expressed by SYNB1618, is dependent on the presence of oxygen^14^. Since oxygen concentrations in the human GI tract are low^15,16^, and our gut-chip studies are performed in the presence of oxygen, LAAD activity in the gut-chip would not be representative of in vivo function. As such, this study utilized the closely related synthetic biotic strain, SYN5183, which is designed to consume Phe only by expressing the enzyme, PAL. The product of PAL activity, TCA, serves as a strain-specific biomarker of Phe consumption that is detectible in plasma (Fig. 2a;^4^) As a biocontainment feature, SYN5183 lacks the *dapA* gene, rendering the strain unable to replicate in the absence of exogenously supplemented diaminopimelic acid (DAP;^4^).

To determine the kinetics of TCA production in a human gut-chip, as well as the relationship between strain activity and dose, SYN5183 was administered in Phe- and DAP-free medium to the gut compartment as a single dose at a low (L; 1.25 × 10^8^ CFU/mL), medium (M; 6.25 × 10^8^ CFU/mL), or high (H; 1.25 × 10^9^ CFU/mL) level (*n* = 6 chips/treatment group). Non-treated chips (NT) served as negative controls. Starting 1 h after SYN5183 inoculation, simulated intestinal fluid (SIF;^17^) containing 5 mM Phe was administered continuously to the gut compartment for 12 h to represent dietary Phe intake (Fig. 2b). Phe dosing levels in the gut-chip were selected based on the design of a previously published non-human primate study, considering differences in available small intestinal absorption surface area^4^. SYN5183 dose concentrations in the chips were selected to represent a range of SYNB1618 doses used in healthy volunteers, considering differences in scale (Clinicaltrials.gov Identifier: NCT03516487). 50–200 µL Phe-free medium was applied to cover the outlet ports of the endothelial (blood) compartment and prevent bubble formation. This resulted in a modest dilution of measured Phe or TCA levels at the 3-h time point (Fig. 2b). Gut and blood compartment effluents were sampled every 3 h to enumerate SYN5183, as well as to determine the concentrations of Phe and TCA by liquid chromatography with tandem mass spectrometry (LC-MS/MS). SYN5183 rapidly cleared the chips and was undetectable in gut compartment effluents 6 h after strain administration for both the Low and Medium dose levels, and after 9 h for the High dose level (Fig. 2c). Administration of SYN5183 resulted in a dose-dependent depletion of Phe in gut compartment effluents (Fig. 2d). High dose SYN5183 significantly reduced gut Phe levels at 3, 6, and 9 h compared to NT controls (*p* < 0.05; 2-way ANOVA, with Tukey’s post-hoc analysis). Low and Medium SYN5183 doses displayed a trend of decreased gut Phe concentrations that did not achieve statistical significance (*p* = 0.067, in comparison to NT). SYN5183 treatment also elevated gut compartment TCA concentrations in a dose-dependent manner for all time points, as compared to negative controls (Fig. 2e). As expected, Phe and TCA concentrations in the gut compartment were time-dependent and associated with the abundance of SYN5183 remaining in the chip (Fig. 2c).

Treatment of PKU patients focuses on the management of blood Phe concentrations^2^. Therefore, a meaningful endpoint for novel therapeutics is the reduction of blood Phe. One unique aspect of the human gut-chip model system is an ability to examine the effects of strain activity within the gut compartment on Phe and TCA concentrations in the endothelial compartment (blood). Co-administration of SYN5183 and Phe to the gut compartment resulted in a dose-dependent increase in TCA concentrations in the blood compartment, indicating transport of this biomarker across the gut barrier (Fig. 2f). TCA concentrations in the blood compartment decreased over time, consistent with the clearance of SYN5183. Untreated control chips exhibited 0.275 ± 0.05 mM Phe (mean ± SEM) in the blood compartment, a value that constitutes mild plasma Phe elevation^2^. Blood compartment Phe concentrations 6 h post-administration were on average 26.9% lower in chips receiving high-dose SYN5183 compared to untreated controls (Fig. 2g), suggesting that strain consumption of dietary Phe can lower systemic Phe concentrations. A significant lowering of blood compartment Phe was observed up to 12 h after SYN5183 administration, despite clearance of the strain by 6–9 h (Fig. 2c). However, a dose-dependent effect of Phe lowering in the blood compartment was not observed. This result is consistent with Phe lowering by SYNB1618 in non-human primates^4^. Importantly, administration of SYN5183 had no significant impact on gut barrier permeability (*P*_app_; Fig. 2h), as measured by the quantification of a fluorescent cascade blue molecule in both compartments over time, indicating that the chips retained a functional gut epithelial barrier.

SYN5183 was constructed using the probiotic chassis strain, *E. coli* Nissle 1917 (EcN), an organism with a long history of safe use in humans. EcN is used in Europe for the treatment of gastrointestinal disorders, including ulcerative colitis, owing in part to its anti-inflammatory characteristics^18^. However, bacterial antigens can also mediate the production of inflammatory cytokines^13^. Human OOC models present an opportunity to study the response of human tissues to synthetic biotics under simulated physiological conditions. To address this, we assayed lactate dehydrogenase (LDH) activity, as an indicator of human cell necrosis and death, in effluents from the gut and blood compartments of chips treated with increasing doses of SYN5183. No significant differences were detected with respect to untreated chips, in either gut or blood compartments (Supplemental Fig. 1a, b). An orthogonal dye exclusion assay conducted using flow cytometry confirmed that SYN5183 had no significant effect on gut (Supplemental Fig. 1c) or endothelial (Supplemental Fig. 1d) cell viability (83 ± 4% and 89 ± 3%, respectively). Gut epithelial stress is often associated with blunted villus structures^13^. Measurements of gut villus height (Supplemental Fig. 1e) revealed no significant difference in villus height associated with SYN5183 administration. Lastly, we assessed the response of human tissues within the chips by performing a Luminex cytokine bead-based enzyme-linked immunosorbent assay (ELISA) on gut compartment effluents. Although SYN5183 administration did not impair gut-blood barrier function, IL-2, IL-7, TNF-α, and IL-21 concentrations were elevated 12 hr post-dose at the high dose level, as compared to NT as indicated (*p* < 0.05, Student’s *t* test with False Discovery Rate correction for multiple hypothesis testing; Supplemental Fig. 1f, Supplemental Table 1). Taken together, these findings demonstrate that a single dose of SYN5183 consumes Phe and produces the biomarker TCA in the human gut-chip model system in a dose-dependent manner, and this activity is associated with a significant lowering of Phe concentrations in the blood compartment but not in a dose-dependent manner. Importantly, no significant effects on human tissue viability, villus architecture, or gut barrier function were observed.

### Continuous exposure to high-dose SYN5183 impairs gut barrier function

**Fig. 3:**
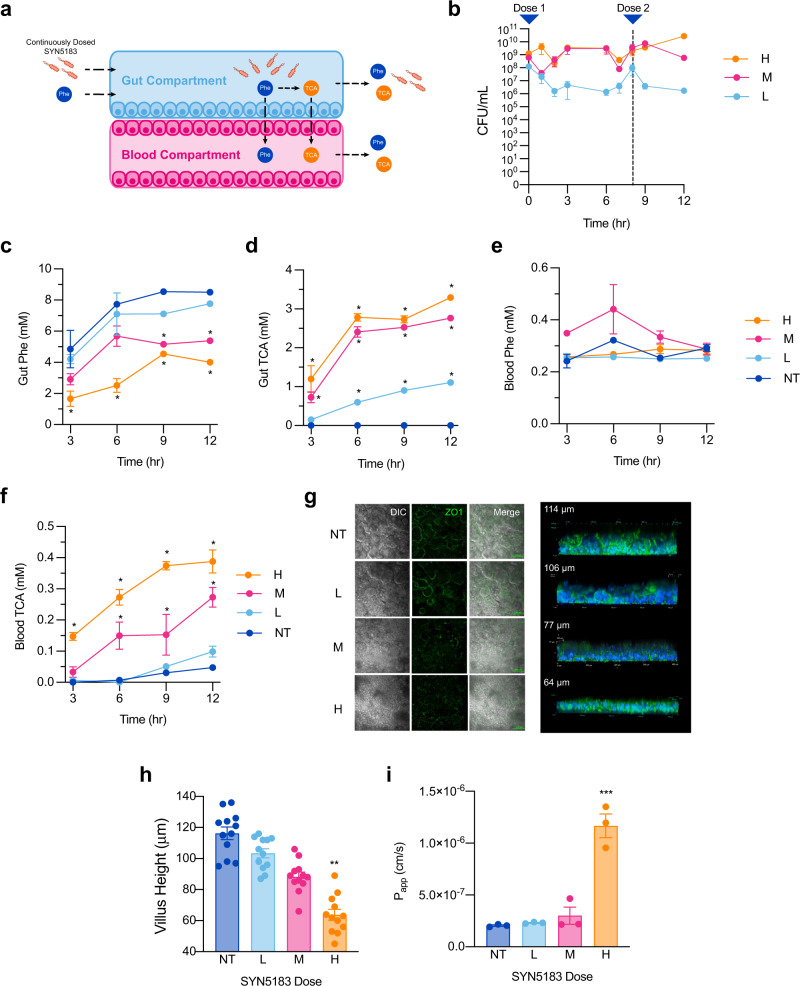
Continuous administration of SYN5183 in a human gut-chip model. **a** Schematic of study design. SYN5183 was administered continuously over 12 h in the gut compartment with 5.0 mM Phe in SIF, SYN5183 CFU were enumerated from gut compartment effluents, and Phe and TCA were quantified using effluents collected from the gut and blood compartments. Fresh, SYN5183-containing inoculum was introduced after 8 h. **b**–**d** Concentrations of SYN5183 (**b**; CFU/mL), Phe (**c**; mM), and TCA (**d**; mM) recovered from gut compartment effluents over time. **e**, **f** Concentrations of Phe (**e**; mM) and TCA (**f**; mM) recovered from blood compartment effluents over time. **g** DIC and ZO-1 (green) staining showed consistent tight-junction formation surrounding macro-villus structures at NT and L—dose and loss of ZO-1 signal and disruption of morphology at M and H—doses, and to the right f-actin (green) staining shows macro-villi structures decreasing in height with increasing dose (*n* = 3 independent gut chips, 4 ROIs per image quantified for villus height). **h**, **i** Villus height (μm) and apparent permeability (*P*_app_; cm/s) across the gut barrier 12 h post-dose. For (**b**–**i**), H (orange), M (pink), and L (light blue) correspond to SYN5183 doses of 1.25 × 10^9^ CFU/mL, 6.25 × 10^8^ CFU/mL, and 1.25 × 10^8^ CFU/mL, respectively. NT (dark blue) corresponds to non-treated (NT) chips. *p < 0.05, **p < 0.01, ***p < 0.01; 2-way ANOVA, with a post-hoc Tukey analysis using a 95% confidence interval compared to the NT group, *n* = 6 gut chips, error bars represent SEM with the middle point representing the mean value. Source data are provided as a Source Data file.

The lack of responses in tissue viability or gut barrier function with administration of a single dose of SYN5183 is consistent with the safety profile of engineered EcN observed in preclinical studies^19^. However, synthetic biotics rapidly transit through the GI tract in preclinical mouse and NHP models^4,19^. To investigate tissue responses during extended exposure to this engineered strain, SIF containing SYN5183 and 5 mM Phe was administered to the gut-chips continuously for 12 h at a low (L; 1.25 × 10^8^ CFU/mL), medium (M; 6.25 × 10^8^ CFU/mL), or high (H; 1.25 × 10^9^ CFU/mL) dose level (*n* = 6 chips/treatment group) (Fig. 3a). Fresh SIF containing SYN5183 was introduced after 6 h, and non-treated chips (NT) served as negative controls. CFU plating of gut compartment effluents confirmed that live SYN5183 was present in the chips throughout this study (Fig. 3b). Continuous SYN5183 administration led to a dose-dependent reduction of gut compartment Phe with concomitant production of TCA (Fig. 3c, d). A dose-dependent increase of TCA was also observed in the blood compartment, though continuous administration of SYN5183 had no significant impact on blood Phe concentrations (Fig. 3e, f).

In contrast to a single dose, continuous administration of SYN5183 resulted in dose-dependent decreases of macro-villus height and ZO-1 staining in the gut-chips (Fig. 3g, h), suggesting reduced function of the gut barrier. Consistent with this observation, continuously administered high-dose SYN5183 also resulted in a statistically significant increase in barrier permeability (Fig. 3i). This effect on barrier permeability may contribute to the lack of blood Phe lowering in the context of continuous strain administration, given the low molecular weight of Phe and the high concentration of Phe administered to the gut compartment. Continuous dosing also had a substantial impact on host cell viability. LDH activity in gut effluents was significantly increased at 6 and 12 h for the high-dose group (*p* < 0.05, 2-way ANOVA, Tukey post-hoc analysis; Supplemental Fig. 2a, b). Flow cytometry conducted at the completion of the study revealed no significant effect on epithelial viability in the gut compartment (*p* = 0.089; ANOVA, Tukey post-hoc analysis Supplemental Fig. 2c). However, a statistically significant decrease in endothelial cell viability was evident in the blood compartment (*p* = 0.031, 2-way ANOVA, Tukey post-hoc analysis; Supplemental Fig. 2d).

In the gut compartment, a dose-dependent increase in IL-2, TNF-α, and IL-15 was evident at 6 h (*p* < 0.05, 2-way ANOVA, Tukey post-hoc analysis; Supplemental Fig. 2e, Supplemental Table 2a). However, IL-15 secretion was markedly decreased for the medium and high dose at 12 h, suggesting that the viability of the host gut cells and breakdown of the gut-blood barrier altered cytokine production and compartment specific concentrations (Supplemental Fig. 2f, Supplemental Table 2b). Conversely, low doses of SYN5183 resulted in significant increases of IL-15, IL-2, and TNF-α. Dose-dependent reduction of TNF-β was also evident in the gut compartment. Interestingly, minimal secreted cytokine activity was evident in the blood compartment, with the exception of the high-dose group at 12 h, where all measured cytokines were significantly elevated by up to 600-fold over NT controls (Supplemental Fig. 2g, h, Supplemental Table 2c, d). To determine whether this markedly increased cytokine secretion in the blood compartment was attributable to bacterial translocation, an additional set of gut-chips (*n* = 5 chips) were continuously dosed with a high dose of SYN5183 (1 × 10^9^ CFU/mL) for 24 h, and SYN5183 CFU was enumerated in gut and blood compartment effluents. While gut effluents harbored the expected concentration of SYN5183, no CFU was detected in the blood compartment (Supplemental Fig. 2i). This result indicates that increased cytokine secretion in the blood compartment is not driven by bacterial translocation. However, it is possible that the transport of bacterial compounds, including lipopolysaccharide (LPS), impacted cytokine expression in the vascular compartment.

### SYN5183 degrades blood Phe in a human gut-chip

**Fig. 4:**
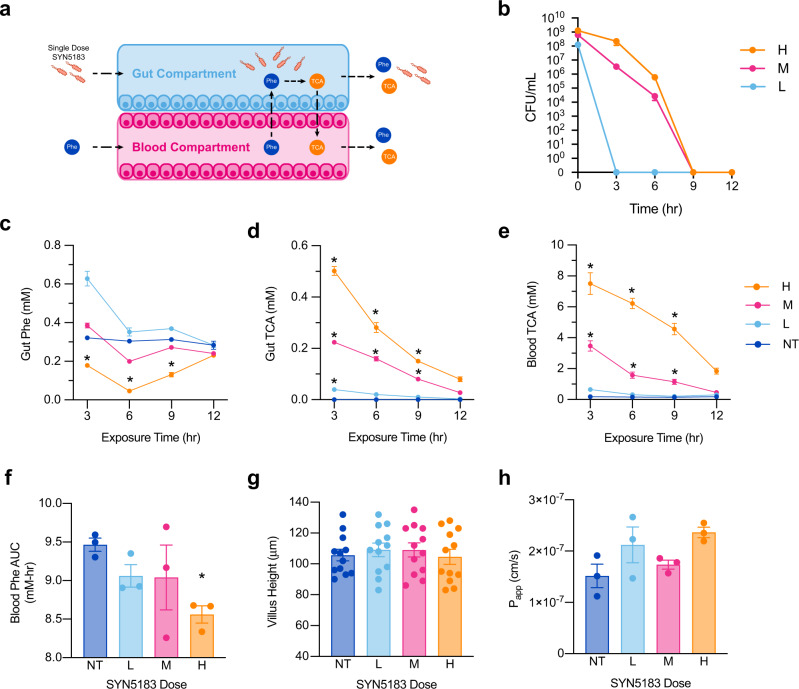
Blood Phe lowering a human gut-chip model with administration of SYN5183. **a** Schematic of study design. A single dose of SYN5183 was administered in the gut compartment, while 1.0 mM Phe was administered continuously to the blood compartment. SYN5183 CFU was enumerated from gut compartment effluents, and Phe and TCA were quantified using effluents collected from the gut and blood compartments. (**b**–**d**, *n* = 3 gut chips) Concentrations of SYN5183 (**b**; CFU/mL), Phe (**c**; mM), and TCA (**d**; mM) recovered from gut compartment effluents over time. (**e**, *n* = 3 gut chips) Concentrations of Phe (mM) recovered from blood compartment effluents over time. **f** Area under the curve (AUC; mM-hr) of Phe recovered from blood compartment effluents over 12 h. (**g**, **h**, *n* = 3 gut chips, villus height measurements were acquired from 4 ROIs per image) Villus height (μm) and apparent permeability (*P*_app_; cm/s) across the gut barrier 12 h post-dose. For (**b**–**h**), H, M, and L correspond to SYN5183 doses of 1.25 × 10^9^ CFU/mL, 6.25 × 10^8^ CFU/mL, and 1.25 × 10^8^ CFU/mL, respectively. NT corresponds to non-treated chips. *p < 0.05; 2-way ANOVA, with a post-hoc Tukey analysis using a 95% confidence interval compared to the NT group, error bars represent SEM with the middle point representing the mean value. Source data are provided as a Source Data file.

In addition to the consumption of Phe from dietary intake, it is important to determine whether a single dose of synthetic biotic can access systemic pools of Phe. There is evidence that plasma Phe can enter the gastrointestinal lumen, through a process termed enterorecirculation^4,20^. Though the specific mechanisms of Phe recirculation are not well defined, this notion is supported by studies in ileostomy patients that demonstrate loss of Phe in gut effluents after being placed on a Phe-free diet^21^. To address this using the human gut- chip model, SYN5183 was administered in Phe-free medium to the gut compartment at a low (L; 1.25 × 10^8^ CFU/mL), medium (M; 6.25 × 10^8^ CFU/mL), or high (H; 1.25 × 10^9^ CFU/mL) dose level (*n* = 3 chips/treatment group). Non-treated chips (NT) served as negative controls. 1 mM Phe was then continuously administered to the blood compartment for 12 h, consistent with levels observed in adult PKU patients without dietary Phe restriction (Fig. 4a;^2^). This procedure resulted in 0.321 ± 0.003 mM Phe (mean ± SEM) available in the gut compartment of untreated chips.

SYN5183 cells cleared the chips within 9 h for all dose levels (Fig. 4b) and demonstrated a dose-dependent lowering of gut compartment Phe with concomitant production of the biomarker TCA (Fig. 4c, d). All concentrations of SYN5183 resulted in significant TCA production at 3 h after strain administration as compared to negative controls, and medium and high doses also achieved statistical significance at 6 and 9 h (Fig. 4d). However, only the high dose of SYN5183 resulted in a statistically significant decrease of gut compartment Phe at 3 h (*p* = 0.021; two-way ANOVA, post-hoc Tukey analysis), 6 h (*p* = 0.002), and 9 h (*p* = 0.025), as compared to NT chips.

In the blood compartment, TCA concentrations were significantly elevated compared to untreated controls at 3, 6, and 9 h for both the medium and high doses of SYN5183 (*p* < 0.05, two-way ANOVA, post-hoc Tukey analysis; Fig. 4e). In addition, the high dose of SYN5183 resulted in a 9.55% decreased area under the curve (AUC) for blood Phe over the course of the study (*p* = 0.032, two-way ANOVA, post-hoc Tukey analysis; Fig. 4f). Importantly, no significant changes in macro-villus height or barrier permeability were observed (Fig. 4g, h), indicating maintenance of a functional gut-endothelial barrier.

Cytokine profiles 12 h post SYN5183 administration (Supplemental Fig. 3, Supplemental Table 3) showed no statistically significant differences in cytokine secretion (*p* < 0.05, Student’s *t* test with False Discovery Rate correction for multiple hypothesis testing). These data suggest that SYN5183 can access circulating Phe and lower systemic concentrations without compromising gut barrier function.

### Computational models to describe Phe kinetics in NHP dosed with SYNB1618

**Fig. 5:**
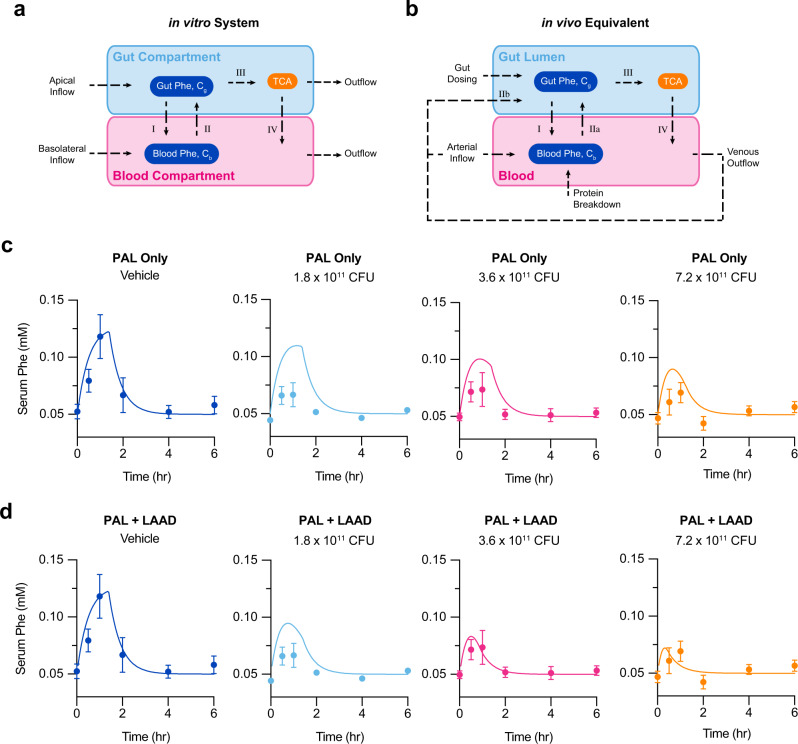
Computational models of Phe kinetics in NHP dosed with SYNB1618. **a**, **b** Schematic representations of Phe kinetics in gut-chips (**a**) and in vivo (**b**). Arrows labeled I-IV represent equivalent processes in the two systems as follows: (I) Uptake of Phe from gut to blood via permeability-surface area product, *PA*, (II) Excretion of Phe from blood to the gut, (III) Metabolism of Phe to TCA by SYN5183 or SYNB1618, normalized to CFU number, and (IV) absorption of TCA from gut to blood via *PA*. For (**b**), the complex in vivo process of hepatic enterorecirculation (IIb) is not implemented in the computational model. Instead, this process is described by direct diffusion from the blood to the gut compartment (IIa), as in the gut chip. **c** Serum Phe concentrations for NHP orally dosed with SYNB1618 and the corresponding in vivo model simulations, assuming PAL activity only. **d** Serum Phe concentrations for NHP orally dosed with SYNB1618 and the corresponding in vivo model simulations, assuming both PAL and LAAD enzyme activity. For (**c**, **d**, in vitro results were acquired from three independent gut chips and extrapolated to published NHP data^4^), points with errors bars indicate experimental data from Isabella et al. ^4^, and solid lines represent simulate data. NHP were dosed at 0 h with a 5.0 g peptone bolus and 0–7.2 × 10^11^ CFU SYNB1618^4^, error bars represent standard deviation with the middle point representing the mean value. Source data are provided as a Source Data file.

Although the gut-chip model emulates the gastrointestinal tract, in vitro systems do not account for all aspects of human physiology. Understanding the translational value of synthetic biotics necessitates the development of mathematical frameworks for integrating data from in vitro and in vivo model systems to predict the behavior of clinical candidates under dynamic conditions of the human gut. We formulated computational models to describe the activity of SYN5183 in gut-chips (Fig. 5a) and in vivo (Fig. 5b, c; see Supplementary Information for a complete description of model development and calibration).

Briefly, the in vitro computational model represents SYN5183 dosing in the gut-chip system (Fig. 5a). Phe consumption by the PAL enzyme, expressed by SYN5183, was assumed to follow Michaelis–Menten kinetics, and kinetic parameters were estimated from in vitro data. Specifically, fitting the in vitro computational model to our gut-chip bolus studies at all SYN5183 dose levels (1.25, 6.25, and 12.5 × 10^8^ CFUs), provided an estimate of *V*_max_ for SYN5183 PAL activity of 0.16 μmol/(10^8^ CFU-h). *K*_*m*_ for the PAL pathway was estimated as 0.2 mM from in vitro Phe consumption assays. This model was also used to estimate a key gut permeability parameter, *P*, by calibration to the in vitro chip studies (*P* = 0.12 cm/h for Phe; *P* = 6 × 10^−4^ cm/h for TCA). Gut wall permeability is an indicator of gut health and a mediator of other physiologically relevant processes, including water flow, motility, and pH.

The in vivo computational model is an extension of the gut-chip computational model that enables time course simulation of blood Phe concentrations after SYN5183 dosing (Fig. 5b). During simulated gastrointestinal transit, Phe is assumed to diffuse into the blood across an effective gut wall surface area, *A* (see Supplementary Information for a discussion on the significance of *A*). Specifically, the model represents the uptake of dietary Phe in the gut according to a permeability-surface area product *PA* (cm^3^/h) and the concentration gradient of Phe between gut and blood compartments. In addition, host tissue protein breakdown, *D* (36 μmol/h in humans;^22^), contributes to blood Phe concentrations, and Phe is assumed to deplete from the blood according to a composite, first-order elimination rate constant, *k* (cm^3^/h). Fasting, steady-state blood Phe levels were used to express *k* in terms of *D*, assuming negligible input of Phe from the gut in the fasting state. Blood Phe levels, *c*_*b*_, are thus represented by Eq. 1:

1 $${V}_{b}\frac{{\rm{d}}{c}_{b}}{{{\rm{d}}t}}={PA}\left({c}_{g}-{c}_{b}\right)+D(1-\frac{{c}_{b}}{{c}_{b,{ss}}})$$

where *c*_*g*_ is the concentration of Phe in the gut, *c*_*b,ss*_ is the steady-state fasting blood Phe concentration, and *V*_*b*_ is the blood volume.

To assess the translational value of the in vivo computational model, time courses of blood Phe concentrations were simulated using this model for comparison to data collected from non-human primates (NHP), orally dosed with the related clinical candidate strain, SYNB1618, and a bolus of peptone (containing approximately 0.25 g Phe) in an earlier study^4^. Simulated dosing comprised a bolus of 0.25 g Phe and engineered cells at four dose levels (0, 1.8 × 10^11^, 3.6 × 10^11^, or 7.2 × 10^11^ CFU). The oral bolus was assumed to pass through the relevant portion of the gut with a transit time τ (h). The transit time, τ, and the effective gastrointestinal surface area, *A*, for NHP were independently fitted to time-course data from the vehicle study arm (0 CFU), providing estimates of 1.4 h, and 20 cm^2^, respectively (see Supplementary Information). The transit time for the bacteria is consistent with passage through a region of the upper GI tract in which the bacterial enzymes are likely to be active, while *A* is a reasonable gut-wall surface area for a 5 ml bolus moving through the small intestine. In all cases, the *V*_max_ for the PAL enzyme determined from the chip studies (0.16 μmol/10^8^ CFU/h) was used.

Simulated serum Phe data are displayed in comparison to the corresponding NHP data in Fig. 5c. The predicted and experimentally measured data show a statistically significant correlation (Pearson’s *R*^2^ = 0.5536, *p* < 0.0001). However, these results reveal that the computational model for SYN5183 activity underestimated serum Phe lowering by SYNB1618 in NHP, particularly at low doses of engineered cells. This may be explained by the expression of an additional Phe-consuming enzyme, LAAD, in the SYNB1618 construct used by Isabella et al., whereas SYN5183 expresses PAL only. To address this, Phe consumption by the LAAD enzyme, expressed by SYNB1618 was incorporated into the computational model (see Supplementary Information), leading to an improved correlation between predicted and experimental data (Pearson’s *R*^2^ = 0.6950, *p* < 0.0001; Fig. 5d). Together, these observations demonstrate that gut-chip model systems, together with physiologically based computational frameworks, can be used to describe the function of synthetic biotics in vivo.

## Discussion

Synthetic biotics represent a new class of living therapeutics with the potential to sense and respond to signals within the human body, to combine therapeutic effectors, and to address the significant unmet medical needs of patients^3^. However, the development of novel therapeutics is both time and resource intensive^3^. Preclinical in vitro and animal models are commonly employed to anticipate the safety and efficacy of drug candidates in humans, but the translational value of these models varies by indication and drug mechanism of action^3,4,12,23^. Ultimately, most drug candidates that do enter clinical efficacy studies fail to meet their primary endpoints and to achieve regulatory approval^4,8,23^. Innovative preclinical models are needed to adequately predict the performance of putative drug candidates in human subjects.

Human OOC technology integrates engineered microfluidic devices and dynamic tissue cultures that emulate organ-level functionality using human cells to bridge the gap between traditional in vitro techniques and animal research, providing enhanced mechanistic insight and relevance to human biology. These platforms enable robust cellular and molecular analysis, fine control over transport and fluid flow dynamics, and incorporation of complex mechanical stimuli that augment the translational value of these laboratory organ models^12,13,24–27^. Gut-chip models, in particular, have shown significant promise in overcoming many limitations of conventional cell culture techniques that fail to capture the complexity and function of human gut tissue. For example, common in vitro model systems of the gut epithelium (e.g., transwell) lack shear stress and fluid flow, which limits the relevance of host-microbe interactions and assessments of barrier function. By contrast, the dynamic culturing conditions in microfluidic systems enhance gut-endothelial barrier health, aid in maintaining microbiome homeostasis, and facilitate nutrient and metabolite transport^13,24,26^. Importantly, gut-chip cultures incorporating both passive and applied mechanical forces develop human gut-like villus projections and an epithelial mucus layer^24,26^. The benefits of dynamic culture also address significant experimental obstacles. For example, these models can prevent bacterial overgrowth of the host cell culture and limit inflammatory responses^13^. This feature of gut-chip models enables a shift of timescale from hours to days and experimental designs that incorporate clinical dosing regimens.

Preclinical models that capture aspects of human gut physiology are critical for interrogating the function of orally administered synthetic biotics, including the diffusion of bacterial products across the gut barrier. In this article, a human gut-chip model system was employed to characterize engineered bacterial strain activity and host tissue responses, as well as to define parameters for computational models that describe strain function in vivo. One limitation of the approach described here is the use of immortalized cell lines. More advanced primary crypt or induced pluripotent stem cell (iPSC) derived human intestinal models have indeed been described that demonstrate significant improvements in capturing host gene expression profiles^8,28,29^. Additional considerations for primary tissue culture models include internal review board governance, donor to donor variability, and increased cost. By contrast, the Caco2 and HT-29 cell lines used in this work have been extensively characterized and are widely available^8,24,25,28,29^. Importantly, Caco2 gut-chip models have proven to be useful for replicating human relevant gut-blood apparent permeability and functional responses. These cell line-based models are suitable for the rapid evaluation of synthetic biotic prototypes in a human gut microenvironment.

SYNB1618 has been previously shown to reduce circulating Phe in an NHP model after an oral Phe bolus and to produce the urinary biomarker, HA, in a dose-dependent manner^4^. In this study, the related strain, SYN5183, demonstrated a dose-dependent production of the strain-specific biomarker, TCA, and reduced blood Phe levels in a human gut-chip by up to 27%. In contrast to static in vitro systems, the dynamic gut-chip model also enabled simulation of bacterial residence time within the gastrointestinal tract, and a single dose of SYN5183 was found to clear the gut-chips within 12 h, leading to concomitant clearance of its product, TCA. Importantly, simultaneous administration of Phe and SYN5183 to the blood and gut compartments, respectively, resulted in a 9.55% cumulative reduction in circulating Phe. These data support the hypothesis that orally administered SYN5183 can consume systemic Phe, in addition to dietary Phe, through a process termed enterorecirculation^4,20^.

In preclinical animal models, engineered EcN strains are well tolerated and rapidly clear the GI tract following oral administration^4,19^. Here, we used the human gut-chip model to examine the effects of synthetic biotic administration at durations of exposure above those that are feasible in vivo by continuous infusion of SYN5183 to the gut compartment. This continuous dosing paradigm (Fig. 3) differs from in vivo exposure to the strain in that this design prevents the rapid bacterial clearance observed in preclinical animal models and humans^4^. In contrast to our studies that simulated in vivo dosing paradigms, we observed that continuous exposure to high-dose SYN5183 in gut-chips decreased macro-villus height and increased barrier permeability. The decreased epithelial surface area associated with villus blunting may alter Phe transport across the gut barrier, consistent with our observation that continuous exposure to SYN5183 abrogated the blood Phe lowering effect observed with clinical dosing designs despite significant differences in gut Phe concentrations. Indeed, villus blunting due to inflammation has been previously shown to impair nutrient transport, barrier maintenance, and mucosal integrity^13,23–27^. These findings suggest that current clinical dosing strategies are preferred, as increased exposure to SYN5183 was not beneficial for lowering circulating Phe.

A notable limitation of our gut-chip model implementation is the presence of ambient oxygen in the system, which contrasts with the low-oxygen concentrations present in the human proximal GI tract^22,30^. Due to this discrepancy, we were unable to directly assess activity of the highly oxygen sensitive LAAD enzyme expressed by the clinical candidate strain SYNB1618^4^. Relatedly, expression of the PheP transporter and PAL enzyme by SYN5183 and SYNB1618 is driven by the low-oxygen responsive FNR promoter and is expected to be maintained in the low-oxygen conditions of the gut. These components are induced during the preparation of biomass (i.e., SYN5183 is “pre-loaded” with PheP and PAL protein). However, continued induction of the FNR promoter in the oxygenated gut-chip model is not expected. This could result in an underestimate of in vivo activity. A complete translational understanding of the potential efficacy of SYNB1618 for PKU patients would require a more thorough examination of LAAD function in a model system that simulates microaerobic conditions. Some groups have indeed described low-oxygen microfluidics implementations for the study of host-bacterial interactions^25^, but such systems are not yet widely available. The development of anaerobic or microaerobic gut-chip models will also be essential for understanding the function of synthetic biotics in the context of a complex endogenous microbial community. The studies described in this work examined SYN5183 in isolation, and a complete understanding of the strain’s function in vivo will require tools that incorporate a representative gut microbiota.

Computational models are powerful tools for embedding experimentally derived parameters in a broader physiological framework. The gut-chip model described here displayed physiologically relevant Phe flux (0.03 nmol/cm^2^*min), indicating that the gut-blood barrier biology was representative of human small intestinal tissue^31,32^. The flux, or the rate of transport through a porous medium, is a measurement of gut-blood barrier. While increases in apparent permeability directly impacts the flux, it is surface area that dictates the magnitude of solute (Phe) transport behavior. The gut-chip represents a microscale anatomical unit of human gut tissue, and thus it is important to consider that the geometry and scale of the model impacts measured values, making computational extrapolation necessary for interpreting in vitro model outcomes. The computational model outlined in this article combines key parameters extracted from gut-chip data with parameters estimating gastrointestinal physiology (e.g., flow rates, tissue volumes, and surface areas), to make predictions of circulating Phe levels in vivo. Using data from the gut-chip system, this model accurately predicted Phe reduction associated with administration of SYNB1618 in NHP.

However, a limitation is that this model implementation does not capture the variability observed in human Phe metabolism (such as residual levels of PAH activity in PKU patients). To capture this, a comprehensive, physiologically based pharmacokinetic (PBPK) model of Phe metabolism is required. Such a model could also include a detailed treatment of enterorecirculation, as well as analysis of engineered strain function within the dynamic and heterogeneous conditions of the human gastrointestinal tract. Advances in OOC technology, such as the incorporation of human induced Pluripotent Stem Cells (iPSCs)^28^, patient-derived primary cell cultures^8,29^, and anaerobic conditions capable of sustaining a human distal gut microbiota^25^, may facilitate these efforts. For example, Novak et al.^33^ recently described a PBPK representation of multiple connected OOC systems, highlighting complex interactions between multiple organ systems^33^.

OOC technology has been successfully applied to the development of other therapeutic modalities. For example, gut-chip analysis of dimethyloxaloylglycine, a small molecule drug candidate for the prevention of radiation-induced gut permeability, predicted clinical outcomes in humans, while these results were not reproduced by rodent models^23^. Furthermore, evaluation of milk oligosaccharides and fermentate from SHIME microbial cultures displayed protein- and metabolite-dependent activation of claudin tight-junctional proteins, limiting small molecule transport as seen in longitudinal human studies involving *B. infantis* treatment of IBD^34,35^. To the best of our knowledge, gut-chip models have not previously been used to validate the in vivo function of synthetic biotics, making this study a foundational case study for evaluating dynamic processes such as strain activity, biomarker production, and host tissue responses, as well as for extrapolation of in vitro data to provide in vivo predictions.

Together, these data indicate that OOC methods, combined with PBPK modeling approaches, provide a powerful set of tools for determining the translational value of engineered bacterial therapeutics. Leveraging this technology has the potential to significantly accelerate the development of synthetic biotics, to enhance probability of success in the clinic, and to address the unmet needs of patients.

## Methods

### Gut-on-a-chip development

The microfluidic device and pressure-driven automated pumping system, collectively termed the Human Emulation System, was procured from Emulate Bio (Boston, MA). The microfluidic device has two compartments separated by a thin, flexible, porous membrane (50 µm thick, 7 µm diameter pores, spaced every 40 µm). The dimensions of the channels are as follows: Upper channel—width = 1 mm, height = 1 mm, culture area 28.0 mm^2^; Lower channel—width = 1 mm, height = 0.2 mm, and culture area 24.5 mm^2^. The microfluidic device and pumping unit are housed and maintained inside a cell culture incubator at 37 °C, 95% relative humidity (RH). Prior to cell seeding, the upper and lower channels were activated with ER-1 and ER-2 surface preparations reagents (Emulate Bio, Boston, MA), and subsequently exposed to UV light for 20 min as directed by the manufacturer. Both channels were flushed twice with 200 µL of phosphate buffer solution (PBS, Gibco) followed by two flushes with cell culture medium to remove any residual ER-1. 40 µL of Geltrex reduced-growth factor basement membrane matrix (Geltrex hESC-Qualified, LDEV, Ready-to-Use, Reduced Growth Factor Basement Membrane Matrix from EHS tumors, Gibco, ThermoFisher Scientific, USA) with an added 100 µg/mL collagen type I (Advanced Biomatrix Inc., USA) was added to the lower and upper compartments and incubated overnight at 4 °C. The following day, channels were flushed with seeding media and prepared for cell seeding. HMVEC, human microvascular endothelial cells (TIME hTERT immortalized, cat #CRL-4045, ATCC, Manassas, VA) were cultured in the lower compartment at a density of 1 × 10^7^ cells/mL (28 µL) in maintenance medium (Vascular Cell Basal Medium ATCC^®^ PCS-100-030 supplemented with a Microvascular Endothelial Growth Kit ATCC® PCS-110-041 without VEGF addition). The microfluidic devices were flipped upside down for 2 h to encourage endothelial adherence to the lower side of the porous membrane. After which the devices were flipped back upright and a 4:1 ratio of Caco2 (Caco2-BBE, ATCC HTB-37, human colorectal carcinoma) and HT29 (HT29-MTX-E12, Millipore-Sigma, 12040401, human adenocarcinoma) gut epithelial cells was added to the upper surface of the membrane at a cell seeding density of 3.75 × 10^6^ cells/mL (40 µL) in experimental medium (DMEM supplemented with 5% fetal bovine serum, ATCC). After 4 h, the microfluidic devices were flushed with fresh degassed medium, docked with medium reservoirs, and maintained within the Human Emulation System. A flow rate of 60 µL/hr was applied to both the upper and lower channels for the entirety of the experiment. The gut-on-a-chip undergoes spontaneous differentiation over a 10-day maturation period, of which cyclical mechanical stretch (10% strain, 0.15 Hz) is applied for the last 7 days to encourage the development of “macro-villus” structures. Nine devices per condition exhibiting macro-villus structures were utilized for experimental testing and evaluation.

### SYN5183 strain construction

SYN5183 was constructed by removing the l-amino acid deaminase (LAAD) gene from SYNB1618 utilizing standard lambda red strain construction methodology^4^. In SYNB1618, LAAD was integrated into the *araC* locus of *E. coli* Nissle 1917, knocking out its natural capability to utilize arabinose for growth. To remove LAAD, we transformed pkd46 into SYNB1618 via electroporation and amplified the original *araC* locus from wild-type Nissle via PCR. This fragment was subsequently used as knock in fragment for lambda red recombination. After electroporation, clones were plated on M9 minimal media plates containing 0.5% arabinose as carbon source. Only cells, with an intact *araC* locus, and therefore LAAD negative, were able to grow under this condition. The removal of LAAD was further verified by PCR and Sanger sequencing. Primers and sequences used for the construction of SYN5183 are provided in Table S4.

### SYN5183 dosing

Frozen glycerol stocks of SYN5183 at 1.65 × 10^11^ CFU/mL were quick thawed diluted in warmed experimental gut medium containing 25% simulated intestinal fluid (full materials and methods located in Supplementary section). Dosages of 1.25 × 10^8^ (Low, L), 6.25 × 10^8^ (Medium, M), or 1.25 × 10^9^ CFU/mL (High, H) of SYN5183 were selected based on in vivo dosing regimens evaluated previously^4^. After the maturation of the gut-on-a-chip, 1 h prior to the start of the experiment, 35 uL of SYN5183 was added to the gut compartment, the chips were then docked with the Pod (cell culture microfluidic reservoir), and held under static, no flow conditions. The 1-h static condition allows the SYN5183 to settle in the chip prior to continuous flow.

### Direct bolus

5 mM phenylalanine (Phe, L-Phenylalanine, Sigma-Aldrich, USA) was added into the experimental medium and continuously flowed through the gut compartment of the microfluidic device for 12 h. All of the effluent was collected every 3 h from both the gut and ‘blood’ compartment outlet waste reservoirs. 200 µL of gut and ‘blood’ compartment inlet medium was collected at 3 h as a reference for permeability and LC-MS/MS analysis.

### Continuous dosing

In addition to the 5 mM phenylalanine the respective dose of SYN5183 was added into the experimental medium and continuously flowed through the gut compartment of the microfluidic device for 12 h. After 6 h a second dose of SYN5183 was added to the inlet reservoir. All of the effluent was collected every 3 h from both the gut and “blood” compartment outlet waste reservoirs. 200 µL of gut and “blood” compartment inlet medium was collected at 3 h as a reference for permeability and LC-MS/MS analysis.

### Phe reduction

Simulation of human circulating levels of blood Phe at 1 mM was added into the inlet of the ‘blood’ compartment of the microfluidic chip. Every 3 h the pass-through effluent from both compartments, and 200 µL from the inlets was collected and stored for future analysis.

### SYN5183 CFU Enumeration

Colonies of SYN5183 from gut-on-a-chip effluent (gut compartment) were plated on LB Agar plates (Animal Free Soytone & Diaminopimelic Acid, Teknova Cat# L1193); 20 µl of effluent was added to 180 µl 1X PBS and serially diluted down to 10^−8^. Five microliters from each dilution were “spot”-plated at each time point in triplicate, and plates were incubated overnight at 37 °C. Colony counts were taken after 18 h (high dose SYN5183 continuous dosing ‘blood’ compartment effluents were analyzed for bacterial presence after 24 h in the gut-chip and showed no countable colonies, further experiments were limited to the gut compartment).

### Cellular morphological and tight-junction analysis

Images of the human gut-on-a-chip villus structures were acquired using differential interface contrast (DIC) and fluorescence (f-actin, ActinGreen 488 (Invitrogen R3110) or ActinRed 555 (Invitrogen R3112) microscopy (Nikon, Ti2 scanning confocal microscope). Gut tissue tight junctions were evaluated using ZO-1 (primary antibody, Invitrogen, 33-9100) immunofluorescence imaging. Gut-chip tissues were washed with phosphate buffered saline (PBS) 2x, followed by fixation in a 4% paraformaldehyde (PFA, Electron Microscopy Sciences, Inc.) phosphate buffer solution at room temperature for 10 min. Following fixation, 0.1% triton-X 100 phosphate buffer solution containing 2.5% bovine serum albumin was added to each channel for 45 min to ensure complete membrane permeabilization and blocking to prevent non-specific immunolabeling. After permeabilization the gut-chip tissue channels were washed with phosphate buffer, and subsequently primary antibody at a concentration of 5 µg/mL was added in permeabilization buffer overnight at 4 °C. After primary antibody labeling, each tissue channel was washed with permeabilization buffer. 5 µg/mL secondary antibody (Goat anti-mouse H &L Alexafluor 488, Invitrogen or Goat anti-mouse H&L AlexaFluor 555, Invitrogen) containing ActinGreen or ActinRed per manufacturers guidelines was added to the respective channels at room temperature for 2 h. Following secondary antibody labeling and actin staining, the tissue channels were flushed with permeabilization buffer and subsequently filled with PBS for confocal imaging. Pipette tips containing excess PBS were placed into the outlet and inlet ports of the gut-chip to prevent channel dehydration. Immunostained gut-chips were stored at 4°C until use. Immunofluorescent cross sections and DIC images were prepared in NIS-Elements Nikon acquisition software. Measurements of villus height and ZO-1 intensity were quantified directly from the images using ImageJ image analysis software.

### Quantification of phenylalanine and trans-cinnamic acid

Quantification of analytes of interest was performed using a triple quadrupole LC-MS/MS Thermo TSQ Quantum Max system. Standards were prepared in water with the following concentrations: 0.032, 0.16, 0.8, 4, 20, 100, and 250 µg/mL. Samples were diluted appropriately with water prior to processing. In a 96-well plate, 10 µL of the standards and samples were transferred, followed by the addition of 90 µL derivatization solution (50 mM of 2-hydrazinoquinoline (2-HQ), dipyridyl disulfide, and triphenylphosphine in acetonitrile with 1 µg/mL of internal standard Phe-^13^C_9_-^15^N). The plate was heat-sealed with a ThermASeal foil (Sigma), mixed, and incubated at 60 °C for 1 h. The samples were then centrifuged at 1000xg for 5 min. To another plate, 20 µL of the derivatized samples were transferred and further diluted with 180 μL of 0.1% formic acid in water/acetonitrile (140:40).

The injection volume used was 10 µL and the run time was 4.25 min at a flow rate of 0.5 mL/min. Mobile phase A was 0.1% formic acid in water and mobile phase B was 0.1% formic acid in acetonitrile/isopropanol (90:10, v/v). Chromatographic separation was carried out using a Phenomenex Luna 5 µm C18 column (3 µm, 100 x 2 mm) with the following gradient: 10% B from 0 to 0.5 min, 10→97% B from 0.5 to 2 min, 97% B from 2 to 4 min, 10% B from 4 to 4.25 min. Multiple reaction monitoring (MRM) in positive mode was used for tandem MS analysis. The following mass transitions were monitored for quantitation: Phe (307.2/186.0), Phe-d_5_ (312.2/186.0), TCA (290.2/131.0), TCA-d_5_ (295.1/138.1), and internal standard Phe-^13^C_9_-^15^N (317.0/186.1).

### Paracellular cascade blue flux assay

Fifty microgram per milliliter of cascade blue (0.59 kDa; ThermoFisher, C687) were introduced to the gut channel (60 mL h^−1^) and fluorescence intensity (390 nm/420 nm) of top and bottom channel effluents were measured using a multi-mode plate reader (BioTek Cytation 5). Apical-to-basolateral flux of the paracellular marker was calculated based on the following equation: *P*_app_=(d*Q*/d*t*)/*A*.d*C*. *P*_app_ (cm s^−1^) denotes the apparent permeability coefficient, d*Q*/d*t* (g s^−1^) is molecular flux, A (cm^2^) is the total area of diffusion and d*C* (mg mL^−1^) is the average gradient. Samples were collected at time 0 from the inlets only, and 12 h from both inlets and outlets of each gut-chip compartment. *P*_app_ values will be calculated by converting relative fluorescence units (RFU) using a standard curve, the mean and standard error will be reported.

### Lactate dehydrogenase (LDH)

Effluent isolated from the outlet reservoirs of six independent chips was added in triplicate to a black-walled, 96-well plate and mixed 1:1 with Cell Membrane Integrity Assay reagent buffer (Promega Inc., USA) and incubated at 37 °C for 2 h as suggested by the manufacturer. The plates were subsequently evaluated using a multi-well spectrophotometer (Cytation 5, Biotek, USA) at an excitation/emission wavelength of 560/590 nm. Lactate dehydrogenase (LDH) is a cytosolic enzyme, upon cell death the cellular membrane becomes compromised and leaks LDH into the cell culture medium. LDH reactivity with the Cell Membrane Integrity Assays provides a direct analysis of LDH concentration.

### Inflammatory cytokine analysis

Secreted inflammatory cytokines were evaluated using a multiplexed bead-based immunoassay (Cytokine 25-Plex Human ProcartaPlex™ Panel 1B, EPX250-12166-901, ThermoFisher Scientific, USA). The following cytokines were evaluated from 3 independent chips in duplicate following the manufacturer’s suggested protocols: GM-CSF; IFN alpha; IFN gamma; IL-1 alpha; IL-1 beta; IL-1RA; IL-2; IL-4; IL-5; IL-6; IL-7; IL-9; IL-10; IL-12 p70; IL-13; IL-15; IL-17A; IL-18; IL-21; IL-22; IL-23; IL-27; IL-31; TNF alpha; TNF beta/LT. Briefly, 50 µL of gut and blood outlet effluent was added to the supplied 96-well plate and mixed with the bead assay. Utilizing the common sandwich enzyme-linked immunosorbent assay (ELISA) approach, pre-mixed beads with capture antibodies for each of the different cytokines were incubated with sample and subsequently detected with a fluorescent conjugated detection antibody. The detected samples isolated on the beads were then fed through an automated, flow-based analyzer (FlexMap 3D, Luminex Inc., USA) and converted to pg/mL using a standard reference curve. ROUT outlier analysis (GraphPad Prism 8) was conducted using a 0.5% false discovery rate as defined by GraphPad Inc identifying outliers with high confidence and removing them for statistical analysis^36^. Averaged and normalized (normalized as fold-change difference with respect to the NT) results are displayed in a heat map to show condition specific differences in secretion. Statistical results are added to the heat maps to show differences with respect to the no treatment control.

### Host cell viability

Gut-chip host cells (Caco2 and HT-29 in the gut compartment, HMVEC in the blood compartment) at the terminal time point (12 h) were taken off of the pumping system and manually flushed with PBS. Trypsin-EDTA was added into each channel to encourage cellular detachment from the ECM coated gut-chip PDMS channels. After a 10-min incubation, using pipetting technique the trypsin solution in each independent channel was mechanically flushed back and forth through the channel at high flow rate to further dislodge the cells. The collected gut or blood compartment cells were washed with DMEM cell culture medium containing 10% FBS, then centrifuged at 300 RCF for 5 min, the supernatant was decanted, and the cells were resuspended in PBS at a concentration of 2 × 10^5^ cells/mL for flow cytometry analysis. Viacount (Millipore Guava Viacount Reagent, Millipore-Sigma, Cat. # 4000-0040) dye exclusion viability assessment reagent was added to the cell-PBS suspension at a 1:1 ratio and incubated at room temperature for 5 min as indicated by the manufacturer’s suggested protocol. Cell viability was determined using flow cytometry (Guava 12-HT Flow Cytometer, Millipore-Sigma), where cells having a compromised cell membrane were labeled with fluorescent Viacount reagent indicating cell death. Using manufacturer settings, optimized gating parameters, and analysis algorithm nucleated viable cells were separated and easily identified from dead cells. 1000 events per sample (*n* = 3) were acquired in duplicate technical measurement indicating a high degree of precision and low error. Duplicates were averaged and the biological mean and standard error of the mean were reported.

### In vitro to in vivo extrapolation of blood Phe levels

Please see Supplementary Information for a full description of computational model development and analysis. Briefly, Berkley Madonna v10, was utilized to solve using iterative methods differential equations representing the in vitro to in vivo extrapolation of gut-chip results.

### Statistics and reproducibility

All results and error bars in this article are represented as mean ± SD. For statistical analyses, a two-way analysis of variance (ANOVA) with Tukey–Kramer multiple comparisons test was performed. Differences between groups were considered statistically significant when *P* < 0.05. GraphPad PRISM 8 (GraphPad, USA) software was utilized to conduct the statistical analysis and produce graphs. Each experimental condition was repeated on independent gut-chips. Microsoft Word, Powerpoint, and Excel softwares (Microsoft, USA) were utilized to organize raw data, format figures, and produce the manuscript. All figures were formatted as vectors in Illustrator (Adobe, USA) for final publication.

### Reporting summary

Further information on research design is available in the Nature Research Reporting Summary linked to this article.

## Supplementary information

Supplementary Information

Reporting Summary

## Data Availability

The datasets and associated source data files generated during and/or analyzed during the current study are available from the corresponding author (mark.nelson.35@us.af.mil) on reasonable request. Source data is available in the Source Data file. Source data are provided with this paper.

## Source data

Source Data

## Code availability

All custom code will be made available by request through the corresponding author (mark.nelson.35@us.af.mil).

## References

[1] de Groot MJ, Hoeksma M, Blau N, Reijngoud DJ, van Spronsen FJ (2010). Pathogenesis of cognitive dysfunction in phenylketonuria: review of hypotheses. Mol. Genet. Metab..

[2] Hanley WB (2004). Adult phenylketonuria. Am. J. Med..

[3] Charbonneau MR, Isabella VM, Li N, Kurtz CB (2020). Developing a new class of engineered live bacterial therapeutics to treat human diseases. Nat. Commun..

[4] Isabella VM (2018). Development of a synthetic live bacterial therapeutic for the human metabolic disease phenylketonuria. Nat. Biotechnol..

[5] Hughes RA, Ellington AD (2017). Synthetic DNA synthesis and assembly: putting the synthetic in synthetic biology. Cold Spring Harb. Perspect. Biol..

[6] Molly K, Vande Woestyne M, Verstraete W (1993). Development of a 5-step multi-chamber reactor as a simulation of the human intestinal microbial ecosystem. Appl. Microbiol. Biotechnol..

[7] Hatton GB, Yadav V, Basit AW, Merchant HA (2015). Animal farm: considerations in animal gastrointestinal physiology and relevance to drug delivery in humans. J. Pharm. Sci..

[8] Kasendra, M. et al. Duodenum Intestine-Chip for preclinical drug assessment in a human relevant model. *Elife* **9**, 10.7554/eLife.50135 (2020). 10.7554/eLife.50135 PMC6959988 31933478

[9] Henry OYF (2017). Organs-on-chips with integrated electrodes for trans-epithelial electrical resistance (TEER) measurements of human epithelial barrier function. Lab Chip.

[10] Huh D, Hamilton GA, Ingber DE (2011). From 3D cell culture to organs-on-chips. Trends Cell Biol..

[11] Kim HJ, Huh D, Hamilton G, Ingber DE (2012). Human gut-on-a-chip inhabited by microbial flora that experiences intestinal peristalsis-like motions and flow. Lab Chip.

[12] Kim HJ, Ingber DE (2013). Gut-on-a-Chip microenvironment induces human intestinal cells to undergo villus differentiation. Integr. Biol..

[13] Kim HJ, Li H, Collins JJ, Ingber DE (2015). Contributions of microbiome and mechanical deformation to intestinal bacterial overgrowth and inflammation in a human gut-on-a-chip. Proc. Natl Acad. Sci..

[14] Motta P, Molla G, Pollegioni L, Nardini M (2016). Structure-function relationships in l-amino acid deaminase, a flavoprotein belonging to a novel class of biotechnologically relevant enzymes. J. Biol. Chem..

[15] Zheng L, Kelly CJ, Colgan SP (2015). Physiologic hypoxia and oxygen homeostasis in the healthy intestine. A review in the theme: cellular responses to hypoxia. Am. J. Physiol. Cell Physiol..

[16] Albenberg L (2014). Correlation between intraluminal oxygen gradient and radial partitioning of intestinal microbiota. Gastroenterology.

[17] Minekus M (2014). A standardised static in vitro digestion method suitable for food—an international consensus. Food Funct..

[18] Scaldaferri F (2016). Role and mechanisms of action of Escherichia coli Nissle 1917 in the maintenance of remission in ulcerative colitis patients: an update. World J. Gastroenterol..

[19] Kurtz CB (2019). An engineered E. coli Nissle improves hyperammonemia and survival in mice and shows dose-dependent exposure in healthy humans. Sci. Transl. Med..

[20] Chang TM, Bourget L, Lister C (1995). A new theory of enterorecirculation of amino acids and its use for depleting unwanted amino acids using oral enzyme-artificial cells, as in removing phenylalanine in phenylketonuria. Artif. Cells Blood Substit. Immobil. Biotechnol..

[21] Fuller MF, Milne A, Harris CI, Reid TM, Keenan R (1994). Amino acid losses in ileostomy fluid on a protein-free diet. Am. J. Clin. Nutr..

[22] Kaufman S (1999). A model of human phenylalanine metabolism in normal subjects and in phenylketonuric patients. Proc. Natl Acad. Sci. USA.

[23] Jalili-Firoozinezhad S (2018). Modeling radiation injury-induced cell death and countermeasure drug responses in a human Gut-on-a-Chip. Cell Death Dis..

[24] Sontheimer-Phelps A (2019). Human colon-on-a-chip enables continuous in vitro analysis of colon mucus layer accumulation and physiology. Cell Mol. Gastroenterol. Hepatol..

[25] Jalili-Firoozinezhad S (2019). A complex human gut microbiome cultured in an anaerobic intestine-on-a-chip. Nat. Biomed. Eng..

[26] Shin W, Hinojosa CD, Ingber DE, Kim HJ (2019). Human intestinal morphogenesis controlled by transepithelial morphogen gradient and flow-dependent physical cues in a microengineered gut-on-a-chip. iScience.

[27] Villenave R (2017). Human gut-on-a-chip supports polarized infection of Coxsackie B1 virus in vitro. PLoS ONE.

[28] Workman MJ (2018). Enhanced utilization of induced pluripotent stem cell–derived human intestinal organoids using microengineered chips. Cell. Mol. Gastroenterol. Hepatol..

[29] Kasendra M (2018). Development of a primary human small intestine-on-a-chip using biopsy-derived organoids. Sci. Rep..

[30] Vockley J (2014). Phenylalanine hydroxylase deficiency: diagnosis and management guideline. Genet. Med..

[31] Adibi SA, Gray SJ (1967). Intestinal absorption of essential amino acids in man. Gastroenterology.

[32] Waisbren SE (2007). Phenylalanine blood levels and clinical outcomes in phenylketonuria: a systematic literature review and meta-analysis. Mol. Genet. Metab..

[33] Novak R (2020). Robotic fluidic coupling and interrogation of multiple vascularized organ chips. Nat. Biomed. Eng..

[34] Kong C (2020). Human milk oligosaccharides mediate the crosstalk between intestinal epithelial Caco-2 cells and lactobacillus PlantarumWCFS1in an in vitro model with intestinal peristaltic shear force. J. Nutr..

[35] Šuligoj, T. et al. Effects of human milk oligosaccharides on the adult gut microbiota and barrier function. *Nutrients* **12**, 10.3390/nu12092808 (2020). 10.3390/nu12092808 PMC7551690 32933181

[36] Motulsky HJ, Brown RE (2006). Detecting outliers when fitting data with nonlinear regression—a new method based on robust nonlinear regression and the false discovery rate. BMC Bioinform..

