# Supplementary material for: Characterization of an engineered live bacterial therapeutic for the treatment of phenylketonuria in a human gut-on-a-chip
Source: Nat Commun. 2021 May 14;12:2805. doi: 10.1038/s41467-021-23072-5 (PMC8121789; doi:10.1038/s41467-021-23072-5)
Supplement: Supplementary file 1 — Supplementary Information [file 41467_2021_23072_MOESM1_ESM.pdf]

Supplementary Information

Characterization of an engineered live bacterial therapeutic for the treatment of phenylketonuria in a human gut-on-a-chip

Authors: M. Tyler Nelson[1,*], Mark R. Charbonneau[2,*], Heidi G. Coia[3], Mary J. Castillo[2], Lt. Corey Holt[1], Eric S. Greenwood[4], Peter J. Robinson[5], Elaine A. Merrill[1], David Lubkowicz[2], Camilla A. Mauzy[1]

* These authors contributed equally

Affiliations: [1]United States Air Force Research Laboratory, 711th Human Performance Wing, Airman Systems Directorate, Bioengineering Division, Wright-Patterson AFB, OH; [2]Synlogic Inc., Cambridge, MA; [3]National Research Council, The National Academies of Sciences, Engineering, and Medicine, Washington D.C.;[4]Oak Ridge Institute for Science and Education, Oak Ridge, TN; [5]The Henry M. Jackson Foundation, Bethesda, MD

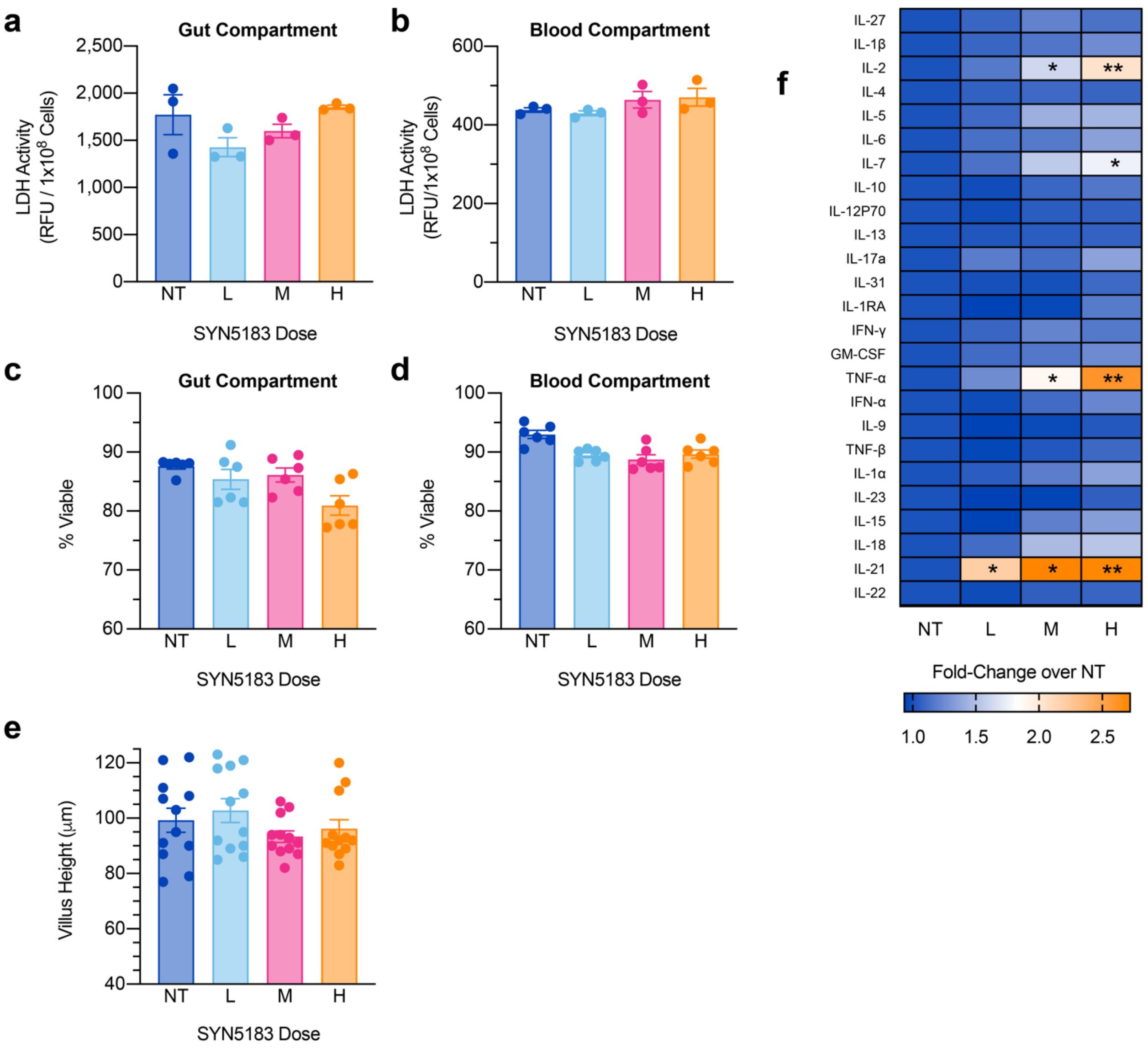

Supplementary Figure 1. Single dose administration of SYN5183 does not alter host cell viability. (a-b) Lactate dehydrogenase (LDH) activity in the gut (a) and blood (b) compartments 12 hours post-dose with SYN5183. (c-d) Host cell viability (%) in the gut (c) and blood (d) compartments 12 hours post-dose with SYN5183. (e) Villus height (µm) 12 hours post-dose (n=3 independent gut-chips, 4 unique regions of interest per chip were measured). (f) Heatmap representation of cytokine concentrations in blood compartment effluents 12 hours post-dose. Values represent fold-change over NT. For (a-f), H, M, and L correspond to SYN5183 doses of 1.25×10^9 CFU/mL, 6.25×10^8 CFU/mL, and 1.25×10^8 CFU/mL, respectively. NT corresponds to non-treated chips. *$p<0.05$, **$p<0.01$; 2-Way ANOVA, post-hoc Tukey analysis for multiple hypothesis testing, compared to the NT group, n = 3 independent gut chips, error bars represent SEM. Source data are provided as a Source Data file.

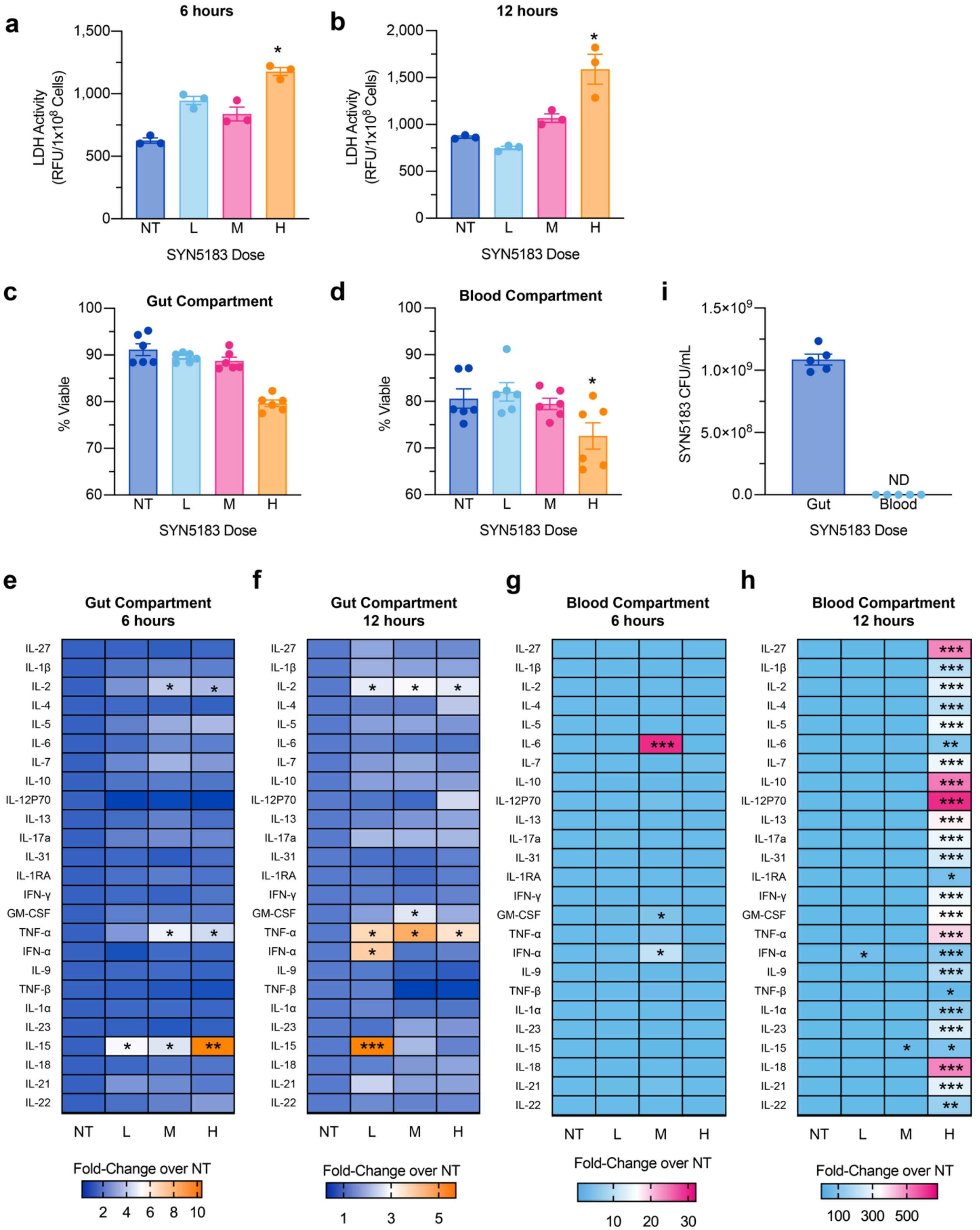
a
6 hours
LDH Activity (RFU/1x10^8 Cells)
SYN5183 Dose
b
12 hours
c
Gut Compartment
% Viable
d
Blood Compartment
i
SYN5183 CFU/mL
ND
Gut
Blood
e
Gut Compartment 6 hours
f
Gut Compartment 12 hours
g
Blood Compartment 6 hours
h
Blood Compartment 12 hours
IL-27
IL-1β
IL-2
IL-4
IL-5
IL-6
IL-7
IL-10
IL-12P70
IL-13
IL-17a
IL-31
IL-1RA
IFN-γ
GM-CSF
TNF-α
IFN-α
IL-9
TNF-β
IL-1α
IL-23
IL-15
IL-18
IL-21
IL-22
NT
L
M
H
Fold-Change over NT

Supplementary Figure 2. Effects of continuous SYN5183 dosing on host cell viability and cytokine expression. (a-b, n = 3 gut chips) Lactate dehydrogenase (LDH) activity in the gut compartment 6 hours (**A**) and 12 hours (**B**) after initiation of dosing with SYN5183. (c-d, n = 3 gut chips) Host cell viability (%) in the gut (c) and blood (d) compartments after 12 hours continuous administration of SYN5183 (n = 3 gut chips with duplicate technical replicates). (e-h) Heatmap representations of cytokine concentrations in the gut compartment after 6 hours (e) and 12 hours (f) and the blood compartment after 6 hours (g) and 12 hours (h) continuous administration with SYN5183. Values represent fold-change over NT. (i) CFU/mL in gut and blood compartment effluents of gut-chips after 24 hr continuous dosing of 1×10^9 CFU/mL SYN5183 (ND: not detected). For (a-h), H, M, and L correspond to SYN5183 doses of 1.25×10^9 CFU/mL, 6.25×10^8 CFU/mL, and 1.25×10^8 CFU/mL, respectively. NT corresponds to non-treated chips. *$p<0.05$, **$p<0.01$, ***$p<0.001$; 2-Way ANOVA, post-hoc Tukey analysis for multiple hypothesis testing, compared to the NT group. ROUT outlier detection method was utilized with a false discovery rate of 0.5%, n = 3 with duplicate technical replicates per condition, error bars represent SEM with the middle point representing the mean value. Source data are provided as a Source Data file.

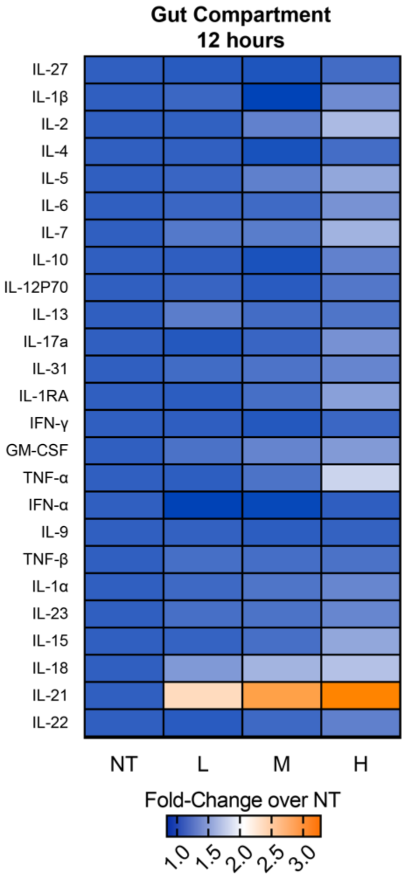

Supplementary Figure 3. Gut compartment cytokine expression in Phe lowering study. Heatmap representation of gut compartment cytokine concentrations 12 hours after single dose administration of SYN5183 with concurrent Phe administration in the blood compartment. H, M, and L correspond to SYN5183 doses of $1.25x10^9$ CFU/mL, $6.25x10^8$ CFU/mL, and $1.25x10^8$ CFU/mL, respectively. NT corresponds to non-treated chips. Values represent fold-change over NT. Source data are provided as a Source Data file.

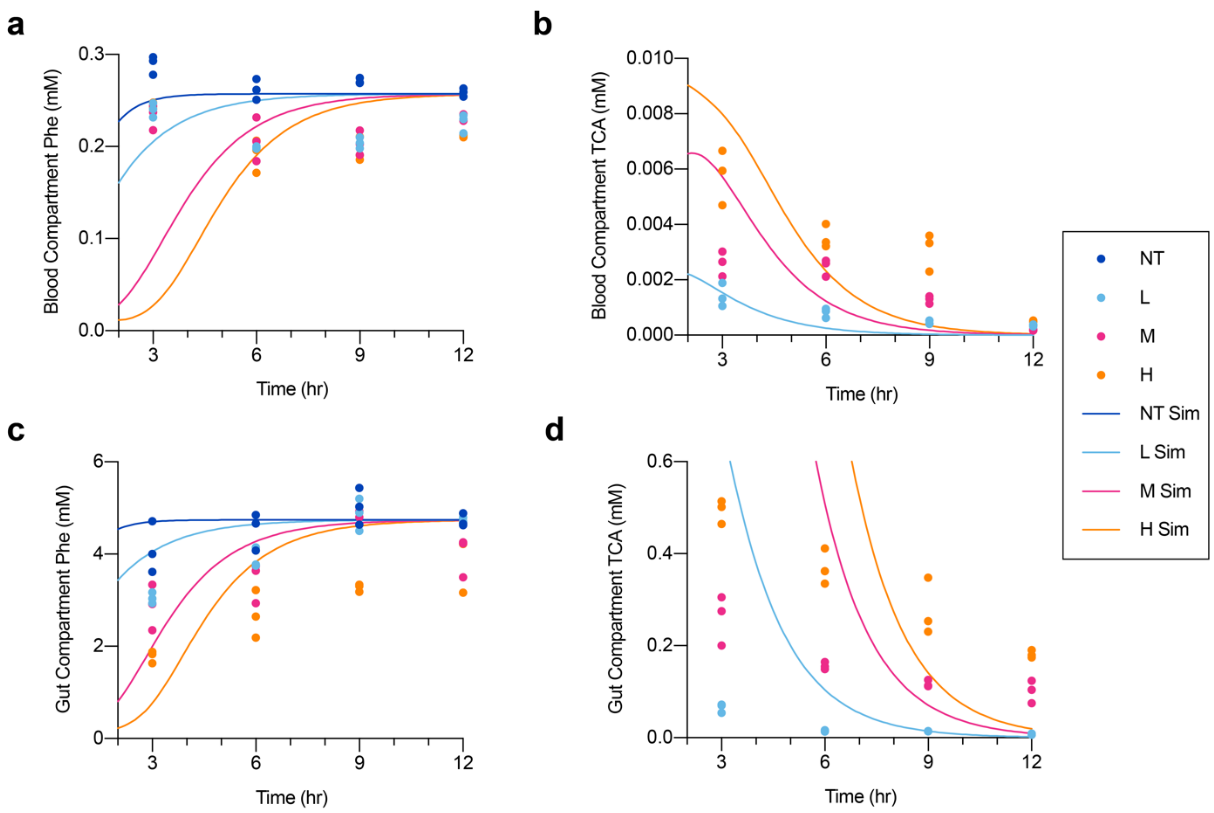

Supplementary Figure 4. Computational simulation of metabolite kinetics in a gut-chip microfluidics model with SYN5183 dosing. Time-course gut-chip and simulated data from continuous infusion of 5 mM Phe into the gut compartment with bolus dosing of SYN5183 for blood compartment Phe (a), blood compartment TCA (b), gut compartment Phe (c), and gut compartment TCA (d). H, M, and L correspond to SYN5183 doses of $1.25x10^9$ CFU/mL, $6.25x10^8$ CFU/mL, and $1.25x10^8$ CFU/mL, respectively. NT corresponds to non-treated chips. Point represents replicate gut-chips and solid lines represent simulated data generated using the *in vitro* model. Fits were used to determine the gut-to-blood permeability P (cm/h) and the SYN5183-mediated maximum metabolic capacity V_{max} (μmol/CFU/h) for PAL activity (values provided in Supplementary **Table 5**). Source data are provided as a Source Data file.

Supplementary Table 1. Cytokine concentrations (pg/mL) in gut-chip blood compartment effluents 12 hours post-dose with single dose administration of SYN5183. H, M, and L correspond to SYN5183 doses of 1.25×10^{9} CFU/mL, 6.25×10^{8} CFU/mL, and 1.25×10^{8} CFU/mL, respectively. NT corresponds to non-treated chips. (Corresponds to Supplementary **Figure 1f**).

**Treatment**	**NT**						**L**						**M**						**H**					
**Cytokine**	**1**	**2**	**3**	**4**	**5**	**6**	**1**	**2**	**3**	**4**	**5**	**6**	**1**	**2**	**3**	**4**	**5**	**6**	**1**	**2**	**3**	**4**	**5**	**6**
IL-27	12.0	8.0	11.0	11.0	8.5	10.0	9.5	11.0	13.0	8.0	13.0	11.0	11.5	12.0	10.5	15.0	12.0	12.0	13.0	12.0	10.0	11.5	13.0	9.0
IL-1β	8.0	8.0	9.0	8.0	9.5	8.0	13.0	8.0	8.0	10.5	7.5	7.5	7.0	12.0	9.5	11.0	10.0	8.5	12.0	9.0	11.0	9.0	12.0	10.0
IL-2	9.0	9.0	6.5	9.0	8.5	9.0	11.5	11.0	9.0	8.5	9.5	10.0	12.5	14.0	15.0	14.0	14.0	13.0	18.0	17.0	17.0	18.0	16.0	18.0
IL-4	18.0	20.0	19.0	21.5	23.0	19.0	22.0	25.5	19.0	18.0	22.0	21.0	21.0	22.0	22.0	23.0	25.0	19.0	23.0	21.0	18.0	22.5	22.0	23.0
IL-5	10.0	10.5	12.0	11.0	15.0	11.0	14.0	14.0	13.0	12.0	9.0	14.0	16.5	17.0	17.0	17.0	14.0	17.0	16.0	19.0	14.0	15.0	16.0	20.0
IL-6	11.5	10.0	10.0	9.0	9.0	8.0	12.0	12.0	12.0	9.0	8.5	11.0	11.0	13.0	10.0	9.0	12.0	12.0	16.0	10.5	12.0	11.0	16.5	11.5
IL-7	24.0	26.0	27.0	28.0	25.5	23.5	32.0	34.0	27.0	25.0	27.0	29.0	36.0	41.0	45.5	41.0	30.0	45.0	39.5	49.0	41.5	48.5	49.5	42.0
IL-10	14.0	14.0	12.0	14.0	14.0	10.0	12.0	13.0	13.0	11.0	12.0	15.0	16.0	15.0	10.0	14.5	15.0	14.0	16.0	15.0	14.0	14.0	16.0	15.0
IL-12p70	26.0	34.0	27.0	28.5	29.0	26.5	26.0	29.0	26.0	32.0	28.5	25.5	28.0	26.0	31.0	28.5	31.5	32.5	35.0	27.5	30.0	30.0	30.0	28.0
IL-13	10.0	12.0	14.5	10.5	15.0	14.0	12.0	14.0	10.5	12.5	14.0	14.0	13.5	15.0	12.0	11.0	14.5	13.0	13.0	15.0	13.0	13.0	14.0	13.0
IL-17a	7.0	8.0	9.5	7.0	8.5	10.5	10.0	11.0	8.0	11.0	9.5	10.0	11.5	9.0	9.5	11.0	7.0	8.0	14.0	11.0	12.0	9.5	10.0	12.0
IL-31	52.0	46.0	46.0	45.0	55.0	49.0	57.0	45.0	47.0	44.5	47.0	49.5	47.0	49.0	46.0	56.0	46.5	45.0	57.0	56.0	50.0	55.0	54.0	48.0
IL-1RA	37.5	41.0	30.5	38.0	31.0	29.0	31.0	37.0	29.5	31.0	30.0	34.5	32.0	32.0	35.0	32.0	31.0	36.5	42.0	36.0	44.5	39.0	41.0	39.5
IFN-γ	10.0	12.0	13.0	10.0	10.0	14.0	13.0	12.0	14.5	12.0	12.0	11.0	15.0	15.0	13.5	15.5	13.0	12.0	11.5	16.0	15.0	9.5	16.0	12.0
GM-CSF	14.0	8.0	11.0	14.0	11.0	9.5	13.0	15.0	11.5	11.0	11.0	13.0	10.5	16.0	11.0	12.0	14.5	14.0	16.0	12.0	15.0	12.0	14.0	15.0
TNF-α	8.0	9.0	7.0	4.0	6.0	6.0	8.0	9.0	8.0	9.0	8.0	8.0	16.5	11.0	9.0	13.5	14.5	10.5	18.0	18.5	16.0	14.0	19.0	18.5
IFN-α	36.0	34.0	40.0	30.0	33.5	35.0	32.0	32.0	37.0	31.0	32.5	38.0	46.0	37.0	28.0	50.0	34.0	35.0	47.0	43.0	39.0	48.0	37.0	41.0
IL-9	43.0	47.0	51.0	40.0	46.0	42.0	40.0	46.5	39.0	40.0	44.0	42.0	42.0	46.0	44.0	48.0	35.0	45.0	53.0	46.0	45.0	42.0	49.5	40.0
TNF-β	73.0	66.0	73.0	81.0	85.0	81.0	76.0	75.0	63.0	65.0	81.0	77.0	75.0	83.0	75.0	88.0	69.0	76.5	82.0	90.0	83.0	81.0	80.5	83.0
IL-1α	45.0	53.5	48.0	48.0	47.0	49.0	52.0	51.0	51.0	48.5	49.0	50.0	63.0	55.0	54.5	55.0	59.0	56.0	70.5	62.0	67.0	64.0	69.0	60.0
IL-23	39.0	33.0	40.0	32.0	33.0	28.0	34.0	29.0	31.0	32.0	34.0	31.0	37.0	34.0	30.0	33.0	31.0	28.0	34.0	40.0	36.0	37.0	36.0	33.0
IL-15	18.0	18.0	19.5	19.0	20.0	17.0	17.0	17.5	19.0	18.0	17.5	15.0	19.0	23.5	26.0	24.0	20.0	20.5	23.5	25.0	25.5	26.0	24.0	24.5
IL-18	7.0	10.5	10.0	10.0	10.0	9.5	12.0	10.0	9.0	13.0	8.0	11.0	15.5	16.5	14.0	10.0	16.0	12.0	12.0	14.5	14.5	15.0	15.0	16.0
IL-21	39.0	40.5	41.0	34.5	36.0	39.0	91.0	90.0	84.0	77.0	85.5	76.0	99.0	105.0	108.0	114.0	105.0	94.0	107.0	103.5	112.0	102.0	98.5	95.5
IL-22	43.0	34.0	41.0	37.5	34.0	41.0	37.0	39.0	37.0	37.0	35.0	39.0	43.0	40.5	41.0	42.0	36.0	40.0	45.0	41.0	39.0	41.0	38.0	44.0

Supplementary Table 2a. Cytokine concentrations (pg/mL) in the gut compartment of gut-chips after 6 hours continuous administration with SYN5183. (Corresponds to **Supplementary Figure 2e**). H, M, and L correspond to SYN5183 doses of $1.25\text{x}10^{9}$ CFU/mL, $6.25\text{x}10^{8}$ CFU/mL, and $1.25\text{x}10^{8}$ CFU/mL, respectively. NT corresponds to non-treated chips. Measurements were taken from 2 independent chips and duplicate technical replicates were analyzed.

**Treatment**	**NT**				**L**				**M**				**H**			
**Cytokine**	**1**	**2**	**3**	**4**	**1**	**2**	**3**	**4**	**1**	**2**	**3**	**4**	**1**	**2**	**3**	**4**
IL-27	25.53	23.33	26.60	21.44	27.54	23.07	25.33	22.63	23.69	22.61	18.58	24.09	27.25	23.41	22.23	35.06
IL-1β	14.21	14.21	19.36	18.75	23.15	17.33	29.53	26.58	26.91	29.02	36.36	30.51	24.76	30.61	29.41	25.29
IL-2	15.90	13.43	14.41	13.99	31.38	26.05	37.72	34.72	53.70	49.45	52.50	57.77	35.15	53.50	55.96	45.67
IL-4	25.71	22.81	24.71	23.80	34.51	25.57	28.83	23.70	20.02	24.20	22.83	37.60	22.12	22.86	22.91	24.05
IL-5	14.51	21.54	14.23	17.74	27.87	26.52	35.84	28.81	48.03	47.94	47.15	51.68	35.97	32.88	110.90	42.57
IL-6	22.56	24.48	22.81	20.07	28.49	26.84	33.14	26.55	27.59	38.79	29.78	85.87	48.69	23.54	36.22	40.20
IL-7	28.57	28.73	20.82	28.88	47.45	46.67	58.86	53.47	78.40	77.52	80.58	87.50	65.75	62.38	60.18	64.05
IL-10	20.29	22.81	21.44	25.55	40.54	26.71	30.55	28.39	33.33	36.80	38.08	37.63	30.34	34.63	36.02	32.06
IL-12p70	38.30	496.46	242.86	31.36	36.32	38.82	57.24	42.84	44.07	158.21	44.35	46.48	35.57	42.24	47.29	47.13
IL-13	22.75	23.55	18.15	18.46	20.55	21.17	21.45	38.30	31.55	30.45	27.81	56.18	24.53	24.21	32.60	29.44
IL-17a	16.15	12.47	14.04	15.53	27.21	29.19	24.66	19.68	29.82	31.32	27.78	29.85	22.75	30.40	30.82	26.30
IL-31	33.52	40.89	46.88	42.73	45.84	44.46	49.77	48.06	33.86	36.24	37.76	34.26	38.02	47.00	46.16	85.93
IL-1RA	93.38	93.35	80.13	104.07	119.99	115.96	149.39	114.80	149.55	150.63	143.93	158.16	114.34	116.86	171.16	111.09
IFN-γ	24.99	24.63	24.68	26.65	40.60	23.85	26.94	29.10	24.93	23.52	27.33	28.70	23.64	27.49	63.60	33.49
GM-CSF	16.29	17.47	18.46	16.95	26.96	24.85	24.73	38.77	27.98	28.71	23.94	31.05	25.90	28.38	27.46	28.18
TNF-α	13.32	12.31	12.36	18.55	34.15	27.50	36.09	33.97	71.56	63.23	74.03	69.54	41.38	70.60	68.36	54.14
IFN-α	140.43	244.04	253.23	579.59	166.24	144.49	208.91	158.59	532.22	523.45	209.24	185.74	254.25	534.85	423.12	138.07
IL-9	39.68	40.99	38.80	40.07	41.52	42.93	46.71	47.78	33.76	78.51	39.77	35.56	33.50	33.96	32.77	60.96
TNF-β	174.63	246.18	188.73	159.52	169.11	168.93	162.92	183.69	130.37	142.89	111.57	163.49	96.09	119.48	103.20	92.27
IL-1α	85.63	82.52	84.74	84.40	89.56	91.42	110.79	92.46	98.54	102.73	98.77	98.21	80.14	99.94	92.54	97.27
IL-23	31.04	38.07	35.86	39.03	33.91	33.18	38.22	31.53	22.90	31.30	25.76	27.07	29.75	29.14	20.13	58.13
IL-15	40.77	150.40	180.83	52.97	657.07	201.14	956.21	357.18	187.54	583.38	550.10	590.50	985.51	1266.29	1280.22	878.38
IL-18	15.08	17.26	16.37	22.47	22.01	20.29	16.46	20.33	28.08	26.60	23.03	26.41	24.41	25.44	28.58	25.27
IL-21	52.26	47.10	37.39	42.21	99.00	100.68	99.79	104.81	94.30	87.75	86.44	84.95	69.90	73.25	63.18	78.06
IL-22	41.62	45.51	47.36	41.29	54.86	55.90	78.46	47.50	95.21	53.28	60.54	101.32	150.46	59.08	68.54	148.86

Supplementary Table 2b. Cytokine concentrations (pg/mL) in the gut compartment of gut-chips after 12 hours continuous administration with SYN5183. (Corresponds to **Supplementary Figure 2f**). H, M, and L correspond to SYN5183 doses of $1.25x10^9$ CFU/mL, $6.25x10^8$ CFU/mL, and $1.25x10^8$ CFU/mL, respectively. NT corresponds to non-treated chips. Measurements were taken from 2 independent chips and duplicate technical replicates were analyzed.

**Treatment**	**NT**				**L**				**M**				**H**			
**Cytokine**	**1**	**2**	**3**	**4**	**1**	**2**	**3**	**4**	**1**	**2**	**3**	**4**	**1**	**2**	**3**	**4**
IL-27	23.61	14.29	22.99	21.24	28.55	40.94	28.14	33.72	27.23	28.26	24.05	20.19	19.09	29.48	25.10	26.18
IL-1β	13.98	17.39	21.10	19.48	27.28	38.97	25.00	34.41	27.35	26.99	32.11	25.87	30.45	29.65	29.56	23.48
IL-2	18.30	14.87	16.42	18.42	38.75	53.98	45.13	44.02	53.95	50.99	46.17	45.78	50.97	44.08	42.98	42.44
IL-4	24.93	22.32	21.87	27.10	30.32	27.29	26.03	22.82	26.34	22.02	25.58	30.09	20.44	24.20	24.18	132.79
IL-5	17.05	32.79	19.91	21.35	29.79	33.43	40.71	34.75	40.70	34.62	37.85	29.67	34.30	31.60	32.41	26.97
IL-6	39.50	14.86	31.76	29.58	34.48	34.19	31.70	32.27	32.02	30.89	24.77	25.88	23.59	27.25	30.25	28.98
IL-7	24.45	53.54	23.15	40.84	41.25	57.69	53.80	50.38	58.28	53.99	52.61	63.64	47.02	49.40	46.07	50.23
IL-10	21.57	22.10	21.38	22.49	30.58	36.27	30.99	34.35	33.53	33.95	30.75	34.89	33.28	37.30	30.49	31.51
IL-12p70	47.11	60.00	42.86	39.57	36.52	50.87	49.06	38.57	36.19	36.74	37.63	34.60	334.74	35.55	36.05	39.66
IL-13	17.61	22.28	24.07	18.89	18.79	21.07	26.07	28.97	26.89	28.48	26.24	32.19	33.04	34.11	34.47	29.41
IL-17a	14.44	13.56	11.76	18.23	26.66	27.98	28.16	27.21	26.92	28.38	25.31	26.28	26.36	26.63	27.88	26.70
IL-31	50.61	23.96	61.11	62.00	37.24	44.79	45.26	46.38	33.28	47.09	46.73	38.52	45.02	49.93	55.02	60.89
IL-1RA	99.50	200.07	121.46	93.46	121.33	138.66	130.66	129.35	102.72	103.64	103.26	109.98	135.41	131.08	127.72	140.05
IFN-γ	24.67	22.64	27.70	27.53	22.74	27.88	29.09	27.60	24.21	21.65	27.18	30.14	23.16	29.70	29.03	33.01
GM-CSF	19.35	17.27	18.87	19.86	27.03	29.13	27.04	30.19	41.24	70.96	41.27	40.00	36.61	33.51	32.66	26.41
TNF-α	12.14	13.44	15.67	14.73	44.89	63.50	56.74	51.88	71.20	72.66	67.77	58.72	59.07	51.29	52.27	39.95
IFN-α	142.20	134.66	379.52	146.67	601.66	1830.70	698.90	176.97	118.29	355.72	125.01	133.80	524.85	119.03	115.78	129.97
IL-9	37.88	59.32	42.50	48.25	50.14	46.15	37.27	50.96	30.15	31.61	32.15	33.99	24.53	29.65	30.40	31.10
TNF-β	202.23	498.55	214.98	220.34	184.18	157.80	174.78	681.66	75.56	99.53	81.56	44.49	70.78	89.29	113.49	79.81
IL-1α	86.82	91.15	92.63	85.24	96.24	100.40	112.42	102.81	77.83	83.82	84.38	80.43	79.75	96.66	79.92	77.97
IL-23	35.85	28.36	34.08	39.15	38.91	23.99	26.03	34.38	96.80	32.80	46.00	40.56	41.54	49.68	49.96	49.58
IL-15	35.88	56.34	119.51	191.47	254.57	533.52	641.16	871.10	106.97	239.38	191.84	218.18	176.48	63.50	82.57	131.89
IL-18	12.43	22.50	17.94	23.02	16.18	19.27	22.24	23.42	25.99	23.68	30.05	27.59	23.11	29.30	29.76	37.49
IL-21	47.47	38.25	39.77	43.16	90.37	98.72	99.63	94.38	59.26	63.91	63.51	70.64	64.23	59.13	67.28	69.87
IL-22	46.62	43.14	47.61	54.72	59.54	49.80	56.80	65.01	52.85	52.54	52.08	60.58	57.39	55.88	50.71	64.17

Supplementary Table 2c. Cytokine concentrations (pg/mL) in the blood compartment of gut-chips after 6 hours continuous administration with SYN5183. (Corresponds to **Supplementary Figure 2g**). H, M, and L correspond to SYN5183 doses of $1.25x10^9$ CFU/mL, $6.25x10^8$ CFU/mL, and $1.25x10^8$ CFU/mL, respectively. NT corresponds to non-treated chips. Measurements were taken from 2 independent chips and duplicate technical replicates were analyzed.

**Treatment**	**NT**				**L**				**M**				**H**			
**Cytokine**	**1**	**2**	**3**	**4**	**1**	**2**	**3**	**4**	**1**	**2**	**3**	**4**	**1**	**2**	**3**	**4**
IL-27	20.00	21.00	17.04	22.86	26.25	18.79	18.08	20.64	23.61	20.23	19.54	15.35	22.72	15.10	27.39	17.46
IL-1β	16.78	17.52	14.65	15.87	14.81	15.49	16.82	15.58	12.03	10.95	14.78	13.90	13.71	15.34	15.57	15.28
IL-2	19.48	14.25	16.64	13.82	13.76	14.14	12.65	14.32	14.94	15.21	20.60	17.27	11.99	12.49	12.67	14.19
IL-4	22.11	25.56	18.89	19.40	16.99	20.71	17.83	17.90	20.45	21.87	20.61	21.69	17.33	22.05	20.89	18.44
IL-5	13.94	15.49	13.03	14.80	15.12	16.08	13.10	15.78	13.79	12.81	18.68	11.55	15.87	13.95	16.21	10.84
IL-6	86.21	18.70	22.32	25.71	33.53	27.96	28.01	27.09	1036.19	1119.65	1769.60	967.05	40.84	23.92	18.62	20.05
IL-7	21.01	22.60	20.74	20.24	20.62	19.95	28.44	26.29	14.37	23.84	23.12	18.97	55.40	16.85	20.66	33.46
IL-10	21.50	23.30	22.28	20.13	21.42	21.20	17.84	19.06	20.30	17.28	21.92	22.11	18.05	19.79	19.20	18.89
IL-12p70	29.54	34.81	34.00	30.76	44.39	29.33	28.48	40.80	64.45	47.98	32.95	56.66	35.11	32.68	32.60	31.28
IL-13	20.16	17.82	16.75	18.85	20.38	19.58	16.42	17.37	19.96	19.98	18.68	14.15	18.34	19.75	13.90	18.69
IL-17a	14.32	12.98	15.15	9.11	19.42	14.92	15.73	14.83	14.60	14.97	10.00	20.37	15.56	13.26	14.54	12.49
IL-31	40.56	45.98	40.24	34.35	29.37	37.29	40.14	40.95	33.48	40.56	35.33	37.50	36.20	40.53	35.87	29.38
IL-1RA	70.44	70.93	71.15	74.80	69.34	74.84	79.84	75.43	74.03	72.44	73.38	80.26	70.67	70.22	72.72	62.01
IFN-γ	21.77	18.96	19.99	22.25	14.44	18.00	21.68	18.15	23.42	17.49	21.33	19.86	18.64	22.32	21.47	19.59
GM-CSF	20.18	17.05	22.03	16.46	45.64	20.51	18.37	14.78	68.83	19.46	82.24	69.31	19.26	12.98	18.09	18.55
TNF-α	11.39	12.40	12.68	10.66	20.24	13.14	10.20	11.14	40.38	11.55	47.95	24.53	12.03	11.56	10.85	10.21
IFN-α	180.44	199.92	139.93	122.48	141.37	323.76	281.61	139.33	1131.89	2041.74	1139.19	1749.45	406.83	136.79	138.44	134.95
IL-9	36.91	27.79	35.26	32.74	25.49	30.31	38.12	31.77	33.23	24.46	29.66	38.07	32.18	32.25	31.77	28.24
TNF-β	191.66	193.56	184.51	190.65	189.92	209.41	166.61	175.34	266.15	217.93	197.76	213.94	201.10	224.99	182.98	189.21
IL-1α	81.26	83.88	78.42	78.78	83.63	90.13	80.08	88.07	70.24	82.75	80.06	85.93	74.53	82.66	73.35	77.83
IL-23	39.66	36.93	32.76	42.20	32.58	31.68	40.93	34.67	33.19	33.22	35.38	39.59	54.17	43.45	34.22	35.87
IL-15	112.41	44.41	144.13	35.70	32.12	72.17	34.38	56.16	38.75	34.82	41.05	34.83	36.81	44.03	45.67	39.38
IL-18	15.55	18.13	11.44	14.62	11.61	16.86	16.72	11.51	16.70	13.19	15.07	14.51	13.30	13.93	13.78	11.68
IL-21	34.96	33.48	33.72	32.39	48.45	41.92	34.29	37.63	44.92	41.06	69.29	58.08	31.53	40.07	34.30	33.22
IL-22	35.25	43.22	37.42	34.53	34.37	37.31	37.81	39.05	45.73	40.75	40.26	36.78	35.46	27.28	36.69	35.16

Supplementary Table 2d. Cytokine concentrations (pg/mL) in the blood compartment of gut-chips after 12 hours continuous administration with SYN5183. (Corresponds to **Supplementary Figure 2h**). H, M, and L correspond to SYN5183 doses of $1.25x10^9$ CFU/mL, $6.25x10^8$ CFU/mL, and $1.25x10^8$ CFU/mL, respectively. NT corresponds to non-treated chips. Measurements were taken from 2 independent chips and duplicate technical replicates were analyzed.

**Treatment**	**NT**				**L**				**M**				**H**			
**Cytokine**	**1**	**2**	**3**	**4**	**1**	**2**	**3**	**4**	**1**	**2**	**3**	**4**	**1**	**2**	**3**	**4**
IL-27	19.54	15.96	40.16	16.07	15.38	14.92	20.08	22.68	18.08	15.29	22.64	19.60	18009.54	22201.53	6932.17	1636.86
IL-1β	13.05	13.22	12.84	12.90	11.38	12.97	12.87	11.20	11.88	17.16	15.88	15.84	1239.65	4431.20	1929.24	1748.93
IL-2	12.25	14.47	11.59	14.62	12.05	13.15	11.54	11.49	9.70	12.05	14.70	13.43	2212.83	4203.71	5125.54	2730.78
IL-4	18.30	21.08	17.19	20.02	24.41	18.64	17.93	17.43	17.23	22.50	18.35	21.28	3322.26	5353.22	3602.52	1476.13
IL-5	12.97	15.44	13.89	11.90	14.84	17.29	14.45	13.27	14.07	14.98	16.23	15.19	3313.61	4129.57	3248.57	5805.59
IL-6	68.41	28.31	26.14	25.14	849.31	22.51	16.32	31.45	233.28	237.11	478.86	541.19	2225.65	3221.48	2448.59	1366.75
IL-7	18.57	12.15	15.67	18.82	17.57	18.27	16.00	17.44	14.19	14.62	20.67	18.71	5515.18	8705.79	2648.98	2733.85
IL-10	20.83	21.69	25.51	32.43	19.88	19.03	21.49	20.26	22.14	24.33	23.76	17.43	11121.19	18453.28	11411.27	14293.93
IL-12p70	28.16	36.91	30.80	33.96	32.78	35.70	28.54	34.82	35.44	35.54	32.51	31.72	20028.38	31577.24	21998.85	14526.50
IL-13	18.96	17.18	20.54	20.09	18.68	17.63	21.32	18.57	18.88	19.37	23.27	16.80	8923.44	10176.57	6175.42	2497.00
IL-17a	12.77	11.50	16.16	11.89	11.55	12.63	12.19	12.13	11.60	16.86	14.47	13.02	2312.94	7879.44	3415.75	1895.24
IL-31	29.57	42.29	33.61	41.85	35.47	40.92	37.48	37.60	42.19	36.68	30.60	37.67	9937.34	16323.36	6251.92	2171.09
IL-1RA	78.59	65.33	62.33	66.96	73.64	78.31	73.11	68.04	70.65	69.73	74.49	76.26	1191.11	4316.92	2968.13	1378.24
IFN-γ	22.04	20.81	18.37	17.04	20.16	17.84	19.00	19.17	21.62	19.32	18.92	17.01	5623.10	8181.98	6002.58	3464.38
GM-CSF	19.05	17.37	17.38	18.47	23.81	18.22	18.18	21.52	29.01	27.74	40.19	43.98	5619.43	10416.08	6426.29	2244.54
TNF-α	11.68	12.71	13.08	9.21	15.30	13.32	11.46	13.31	17.52	13.34	15.92	20.41	3449.59	6138.15	5959.28	3513.10
IFN-α	148.75	149.92	144.26	164.16	1710.99	4471.94	2985.07	7134.82	824.49	1591.90	1422.02	1150.73	9246.35	17139.65	10130.29	5063.99
IL-9	29.14	26.68	31.35	31.76	35.64	33.34	30.58	32.50	31.14	28.99	29.34	30.55	3770.78	8254.08	6229.11	1574.57
TNF-β	225.43	222.75	228.16	186.91	194.52	190.49	214.17	192.24	186.14	224.61	169.27	182.72	1188.20	2522.63	1345.16	1512.22
IL-1α	81.03	87.62	84.81	78.73	81.11	79.25	88.30	80.55	81.04	75.07	159.90	78.96	4383.19	13631.18	9327.15	3528.00
IL-23	38.61	26.27	40.82	36.11	38.42	38.82	41.17	39.68	31.72	31.63	743.48	34.66	9033.30	14428.88	7235.22	4525.04
IL-15	28.26	295.29	419.59	32.87	43.15	51.86	475.50	664.50	7137.28	702.49	215.56	2232.66	10088.99	16914.88	10702.17	3885.15
IL-18	13.44	13.17	13.65	12.70	14.52	17.42	13.95	17.89	12.81	13.46	13.72	13.88	6616.21	8893.49	7651.16	5152.22
IL-21	35.88	37.98	33.25	34.70	48.41	40.02	35.77	69.47	36.92	34.76	43.07	35.75	10031.27	14982.75	8949.26	3923.57
IL-22	41.97	44.50	42.13	35.62	38.01	36.16	37.15	31.11	31.41	33.94	35.52	38.66	4450.27	6799.03	4471.11	2111.34

Supplementary Table 3. Gut compartment cytokine concentrations in Phe lowering study 12 hours after single dose administration of SYN5183 with concurrent Phe administration in the blood compartment. H, M, and L correspond to SYN5183 doses of 1.25x10^9 CFU/mL, 6.25x10^8 CFU/mL, and 1.25x10^8 CFU/mL, respectively. NT corresponds to non-treated chips. (Corresponds to **Supplementary Figure 3**).

**Treatment**	**NT**						**L**						**M**						**H**					
**Cytokine**	**1**	**2**	**3**	**4**	**5**	**6**	**1**	**2**	**3**	**4**	**5**	**6**	**1**	**2**	**3**	**4**	**5**	**6**	**1**	**2**	**3**	**4**	**5**	**6**
IL-27	15.5	17.0	10.0	12.0	11.0	10.5	12.0	12.0	12.0	12.0	12.0	14.0	13.0	14.0	12.0	13.0	7.5	12.0	14.0	11.0	13.0	18.0	11.5	14.0
IL-1β	13.0	10.0	10.5	10.5	12.0	13.0	11.0	8.5	14.0	14.0	15.5	9.0	11.0	11.0	7.0	11.0	9.0	9.0	15.0	13.0	9.5	17.0	17.0	16.0
IL-2	9.0	9.0	11.0	12.0	11.0	11.5	9.0	11.0	7.0	12.0	13.0	12.0	14.0	12.0	12.5	17.0	11.5	9.5	14.0	20.0	13.5	19.5	15.0	18.0
IL-4	24.0	24.0	26.0	25.0	27.0	21.0	25.0	23.5	22.0	31.0	22.0	24.5	19.0	22.0	24.0	22.0	21.0	27.5	32.0	25.5	23.5	20.0	30.0	27.5
IL-5	13.0	11.5	12.0	13.0	9.0	13.5	13.0	13.0	12.0	14.0	12.0	10.5	11.5	16.0	12.5	14.0	18.0	14.0	21.0	21.0	10.0	15.5	16.0	20.5
IL-6	14.0	12.0	13.0	11.5	12.0	12.0	13.0	11.0	12.0	17.5	12.5	11.5	13.0	11.0	15.0	14.0	15.0	11.0	15.0	13.0	17.0	19.5	16.0	17.0
IL-7	27.0	30.0	23.5	28.0	24.0	25.0	33.0	33.0	28.0	27.0	30.0	29.5	28.5	38.0	28.0	28.5	28.0	33.5	43.0	37.0	45.0	41.5	35.5	38.0
IL-10	18.0	20.0	18.0	19.0	18.5	17.0	20.5	19.0	17.0	17.0	19.0	17.5	17.0	18.0	18.0	16.0	16.0	17.0	24.0	26.0	23.0	18.0	20.0	22.0
IL-12p70	29.0	34.0	26.5	33.5	37.0	34.0	35.5	31.0	32.0	35.0	33.0	33.0	34.0	35.0	31.0	27.5	30.0	32.0	37.0	35.5	34.5	38.5	35.0	40.5
IL-13	13.0	17.0	14.0	13.0	16.0	11.0	15.5	14.0	16.0	18.0	18.0	17.0	16.0	14.0	13.5	13.5	17.0	16.0	14.0	16.0	15.0	15.5	17.0	17.0
IL-17a	12.0	11.0	12.0	10.0	11.0	13.0	12.0	12.0	8.0	12.0	12.0	10.0	9.0	12.0	10.0	15.5	11.0	14.0	13.0	12.0	14.0	16.0	17.0	18.0
IL-31	57.5	58.0	55.0	57.0	56.0	54.0	67.0	59.0	56.5	56.5	59.0	63.5	60.5	61.0	66.0	66.0	57.0	66.0	67.0	70.0	73.0	69.5	61.0	74.0
IL-1RA	35.0	37.0	42.0	41.0	35.0	39.0	39.0	35.0	42.0	38.0	36.0	36.0	36.0	44.0	36.0	37.0	52.5	44.0	55.0	50.5	57.0	48.0	54.0	56.0
IFN-γ	18.0	16.0	13.0	17.0	16.0	18.0	15.0	16.0	18.5	18.0	16.0	14.0	13.5	18.5	14.0	15.0	15.0	18.0	17.0	15.5	17.0	14.0	17.0	22.0
GM-CSF	15.0	14.0	11.0	12.0	14.0	13.0	16.0	16.5	17.0	13.0	11.0	14.0	13.0	16.0	14.5	22.0	15.0	16.0	16.0	18.0	15.0	18.5	19.0	21.0
TNF-α	8.0	11.0	7.0	9.0	11.0	11.0	7.0	10.0	10.5	9.0	11.0	9.0	6.0	11.5	9.0	10.0	14.0	13.0	17.0	18.0	18.0	16.0	15.0	16.0
IFN-α	54.0	68.0	55.0	61.5	65.5	57.0	43.0	49.5	46.5	48.5	59.0	55.0	56.0	48.0	60.0	59.0	40.0	49.0	57.0	56.0	59.0	68.0	63.0	56.0
IL-9	60.0	66.0	63.0	68.0	72.0	70.0	67.0	66.0	64.0	75.0	68.0	64.0	59.0	67.0	73.0	66.5	66.0	63.0	71.0	68.0	72.0	61.0	66.0	67.0
TNF-β	98.0	97.0	90.0	95.0	93.0	93.0	112.0	104.0	108.0	101.0	93.5	98.0	93.5	109.0	98.5	112.0	105.0	95.5	97.0	112.0	111.5	104.5	101.0	102.0
IL-1α	61.0	67.0	60.5	72.0	64.0	65.0	69.0	72.0	62.0	66.0	77.0	65.0	77.0	71.0	71.5	77.0	64.0	79.0	78.0	73.0	87.0	77.0	77.0	88.0
IL-23	38.0	42.5	40.5	37.5	37.0	36.0	46.5	46.5	45.0	39.0	35.0	41.0	42.5	43.0	41.0	47.0	39.5	45.0	44.0	46.5	51.0	57.0	42.0	45.0
IL-15	18.0	21.5	20.5	17.0	20.0	22.5	19.0	23.0	18.0	20.0	22.0	20.0	20.0	23.0	20.0	27.0	21.5	19.0	28.0	25.5	27.0	32.0	28.5	31.5
IL-18	9.0	12.5	9.5	11.0	11.0	10.5	13.0	16.5	13.5	14.0	15.0	14.0	14.0	17.0	17.5	12.0	20.0	17.0	18.5	14.0	13.5	22.0	17.0	18.0
IL-21	56.0	52.0	44.5	54.0	48.5	45.0	125.5	118.0	119.0	119.0	125.0	119.0	144.0	147.0	148.0	148.0	151.0	161.0	166.0	145.0	171.0	170.0	160.0	170.0
IL-22	50.5	58.0	57.0	61.5	53.0	64.0	64.0	58.0	51.0	53.0	57.0	53.0	52.0	62.0	60.0	65.0	54.0	70.0	66.0	66.0	71.0	71.0	65.5	73.0

Supplementary Table 4. Primers and Sequences used for the construction of SYN5183 from SYNB1618.

Component	Sequence 5 ' to 3'	Comments
SYNB1618 araBC – LAAD chromosomal sequence	**ACAACCTGCCCTAAACTCGC**TCGGACTCGCCCCGGTGCATTTTTAAATACTCGCGAGAAATAGAGTTGATCGTCAAAACCGACATTGCGACCGACGGTGGCGATAGGCATCCG<br>GGTGGTGCTCAAAAGCAGCTTCGCCTGACTGATGCGCTGGTCCTCGCGCCAGCTTAATACGCTAATCCCTAACTGCTGGCGGAACAAATGCGACAGACGCGACGGCGACAGGCA<br>GACATGCTGTGCGACGCTGGCGATATCAAAATTACTGTCTGCCAGGTGATCGCTGATGTACTGACAAGCCTCGCGTACCCGATTATCCATCGGTGGATGGAGCGACTCGTTAATC<br>GCTTCCATGCGCCGCAGTAACAATTGCTCAAGCAGATTTATCGCCAGCAATTCCGAATAGCGCCCTTCCCCTTGTCCGGCATTAATGATTTGCCCAAACAGGTCGCTGAAATGCGG<br>CTGGTGCGCTTCATCCGGGCGAAAGAAACCGGTATTGGCAAATATCGACGGCCAGTTAAGCCATTCATGCCAGTAGGCGCGCGGACGAAAGTAAACCCACTGGTGATACCATTC<br>GTGAGCCTCCGGATGACGACCGTAGTGATGAATCTCTCCAGGCGGGAACAGCAAAATATCACCCGGTCGGCAGACAAATTCTCGTCCCTGATTTTTCACCACCCCCTGACCGCGA<br>ATGGTGAGATTGAGAATATAACCTTTCATTCCCAGCGGTCGGTCGATAAAAAAATCGAGATAACCGTTGGCCTCAATCGGCGTTAAACCCGCCACCAGATGGGCGTTAAACGAGT<br>ATCCCGGCAGCAGGGGATCATTTTGCGCTTCAGCCATACTTTTCATACTCCCGCCATTCAGAGAAGAAACCAATTGTCCATATTGCATCAGACATTGCCGTCACTGCGTCTTTTACT<br>GGCTCTTCTCGCTAACCCAACCGGTAACCCCGCTTATTAAAAGCATTCTGTAACAAAGCGGGACCAAAGCCATGACAAAAACGCGTAACAAAAGTGTCTATAATCACGGCAGAA<br>AAGTCCACATTGATTATTTGCACGGCGTCACACTTTGCTATGCCATAGCATTTTTATCCATAAGATTAGCGGATCCAGCCTGACGCTTTTTTTCGCAACTCTCTACTGTTTCTCCATA<br>CCTCTAGAAATAATTTTGTTTAACTTTAAGAAGGAGATATACAT*ATGAACATTTCAAGGAGAAAGCTACTTTTAGGTGTTGGTGCTGCGGGCGTTTTAGCAGGTGGTGCGGCTTTAGTTCC*<br>*AATGGTTCGCCGTGACGGCAAATTTGTGGAAGCTAAATCAAGAGCATCATTTGTTGAAGGTACGCAAGGGGCTCTTCCTAAAGAAGCAGATGTAGTGATTATTGGTGCCGGTATTCAAGGGAT*<br>*CATGACCGCTATTAACCTTGCTGAACGTGGTATGAGTGTCACTATCTTAGAAAAGGGTCAGATTGCCGGTGAGCAATCAGGCCGTGCATACAGCCAAATTATTAGTTACCAAACATCGCCAGAA*<br>*ATCTTCCCATTACACCATTATGGGAAAATATTATGGCGTGGCATGAATGAGAAAATTGGTGCGGATACCAGTTATCGTACTCAAGGTCGTGTAGAAGCGCTGGCAGATGAAAAAGCATTAGATA*<br>*AAGCTCAAGCGTGGATCAAAACAGCTAAAGAAGCGGCAGGTTTTGATACACCATTAAATACTCGCATCATTAAAGGTGAAGAGCTATCAAATCGCTTAGTCGGTGCTCAAACGCCATGGACTGT*<br>*TGCTGCATTTGAAGAAGATTCAGGCTCTGTTGATCCTGAAACAGGCACACCTGCACTCGCTCGTTATGCCAAACAAATCGGTGTGAAAATTTATACCAACTGTGCAGTAAGAGGTATTGAAACT*<br>*GCGGGTGGTAAAATCTCTGATGTGGTGAGTGAGAAAGGGGCGATTAAAACGTCTCAAGTTGTACTCGCTGGGGGTATCTGGTCGCGTTTATTTATGGGCAATATGGGTATTGATATCCCAACG*<br>*CTCAATGTATATCTATCACAACAACGTGTCTCAGGGGTTCCTGGTGCACCACGTGGTAATGTGCATTTACCTAATGGTATTCATTTCCGCGAACAAGCGGATGGTACTTATGCCGTTGCACCAC*<br>*GTATCTTTACGAGTTCAATAGTCAAAGATAGCTTCCTGCTAGGGCCTAAATTTATGCACTTATTAGGTGGCGGAGAGTTACCGTTGGAATTCTCTATTGGTGAAGATCTATTTAATTCATTTAAAA*<br>*TGCCGACCTCTTGGAATTTAGATGAAAAAACACCATTCGAACAATTCCGAGTTGCCACGGCAACACAAAATACGCAACACTTAGATGCTGTTTTCCAAAGAATGAAAACAGAATTCCCAGTATTT*<br>*GAAAAATCAGAAGTTGTTGAACGTTGGGGTGCCGTTGTGAGTCCAACATTTGATGAATTACCTATCATTTCTGAGGTCAAAGAATACCCAGGCTTAGTGATTAACACGGCAACAGTGTGGGGTA*<br>*TGACAGAAGGCCCGGCAGCGGGTGAAGTGACCGCTGATATTGTCATGGGCAAGAAACCTGTTATTGATCCAACGCCGTTTAGTTTGGATCGTTTTAAGAAGTAA*GGAATCGACTCCACGTCC<br>CTAGCGTGTGTAGGCTGGAGCTGCTTCGAAGTTCCTATACTTTCTAGAGAATAGGAACTTCGGAATAGGAACTAAGGAGGATATTCATATGGTTGAGTCATCCGCAGAAGAGACG<br>AGGTATTAGAAGCCAACCTGGCGCTGCCAAAACACAACCTGGTCACGCTCACCTGGGGCAATGTCAGCGCCGTTGATCGCGGGCGCGGCGTCCTGGTGATCAAACCTTCCGGCGT<br>CGACTACAGCATCATGACCGCTGACGATATGGTCGTGGTCAGCATCGAAACCGGTGAAGTGGTTGAAGGTACGAAAAAGCCCTCCTCCGACACGCCAACTCACCGGCTGCTCTAT<br>CAGGCATTCCCGTCTATTGGCGGCATTGTGCACACACACTCGCGCCACGCCACCATCTGGGCGCAGGCGGGCCAGTCGATTCCAGCAGCCGGCACCACCCACGCCGACTATTTCT<br>ACGGCACCATTCCCTGCACCCGCAAAATGACCGACGCAGAAATCAACGGTGAATATGAGTGGGAAACCGGTAACGTCATCGTAGAAACCTTCGAAAAACAGGGTATCAATGCAG<br>CGCAAATGCCCGGCGTGCTGGTCCATTCTCACGGCCCATTTGCATGGGGAAAAAACGCCGAAGATGCGGTGCATAACGCCATCGTGCTGGAAGAAGTCGCTTATATGGGGATATT<br>CTGCCGTCAGTTAGCGCCGCAGTTACCGGATATGCAGCAAACGCTGCTGGATAAACACTATCTGCGTAAGCATGGCGCGAAGGCATATTACGGGCAGTAATGACTGTATAAAACC<br>ACAGCCAATCAAACGAAACCAGGCTATAATCAAGCCTGGTTTTTTATTGGATTTTCAGCGTGGCGCAGGCAGGTTTTATCTTAACCCGACACTGGCGGGACACCCCGCAAGGAAC<br>CGAAGTTTCCTTTTGGTTAGCGACGGACAACGGGCCGTTGCAGGTTACGCTTGCGCCGCAAGAGTCCGTGGCGTTTATTCCCGCCGATCAGGTTCCCCGCGCCCAGCATATTTGC<br>AGGGTGAGCAAGGCTTTCGCCTGACGCCGCTGGCGTTAAAGGATTTTCATCGCCAGCCGGTGTATGGCCTTTACTGTCGCGCCCATCGCCAGTTGATGAATTACGAAAAGCGCCT<br>GCGTGAAGCTGGCGTTACCGTCTACGAGGCCGATGTGCGCCCGCCAGAACGCTATCTGATGGAGCGGTTTATCACCTCGCCAGTGTG**GGTCGAGGGTGATATGCGC**	Primer binding sequence in **bold**. LAAD coding sequence in *italic*.
Primer Ara-F	ACAACCTGCCCTAAACTCGC	Forward primer used to amplify the wild type araBC locus from EcN
Primer Ara-R	GCGCATATCACCCTCGACC	Reverse primer used to amplify the wild type araBC locus from EcN
SYN5183 – araBC sequence (LAAD removed)	**ACAACCTGCCCTAAACTCGC**TCGGACTCGCCCCGGTGCATTTTTAAATACTCGCGAGAAATAGAGTTGATCGTCAAAACCGACATTGCGACCGACGGTGGCGATAGGCATCCG<br>GGTGGTGCTCAAAAGCAGCTTCGCCTGACTGATGCGCTGGTCCTCGCGCCAGCTTAATACGCTAATCCCTAACTGCTGGCGGAACAAATGCGACAGACGCGACGGCGACAGGCA<br>GACATGCTGTGCGACGCTGGCGATATCAAAATTACTGTCTGCCAGGTGATCGCTGATGTACTGACAAGCCTCGCGTACCCGATTATCCATCGGTGGATGGAGCGACTCGTTAATC<br>GCTTCCATGCGCCGCAGTAACAATTGCTCAAGCAGATTTATCGCCAGCAATTCCGAATAGCGCCCTTCCCCTTGTCCGGCATTAATGATTTGCCCAAACAGGTCGCTGAAATGCGG<br>CTGGTGCGCTTCATCCGGGCGAAAGAAACCGGTATTGGCAAATATCGACGGCCAGTTAAGCCATTCATGCCAGTAGGCGCGCGGACGAAAGTAAACCCACTGGTGATACCATTC<br>GTGAGCCTCCGGATGACGACCGTAGTGATGAATCTCTCCAGGCGGGAACAGCAAAATATCACCCGGTCGGCAGACAAATTCTCGTCCCTGATTTTTCACCACCCCCTGACCGCGA<br>ATGGTGAGATTGAGAATATAACCTTTCATTCCCAGCGGTCGGTCGATAAAAAAATCGAGATAACCGTTGGCCTCAATCGGCGTTAAACCCGCCACCAGATGGGCGTTAAACGAGT<br>ATCCCGGCAGCAGGGGATCATTTTGCGCTTCAGCCATACTTTTCATACTCCCGCCATTCAGAGAAGAAACCAATTGTCCATATTGCATCAGACATTGCCGTCACTGCGTCTTTTACT<br>GGCTCTTCTCGCTAACCCAACCGGTAACCCCGCTTATTAAAAGCATTCTGTAACAAAGCGGGACCAAAGCCATGACAAAAACGCGTAACAAAAGTGTCTATAATCACGGCAGAA<br>AAGTCCACATTGATTATTTGCACGGCGTCACACTTTGCTATGCCATAGCATTTTTATCCATAAGATTAGCGGATCCAGCCTGACGCTTTTTTTCGCAACTCTCTACTGTTTCTCCATA<br>CCCGTTTTTTTGGATGGAGTGAAACGATGGCGATTGCAATTGGCCTCGATTTTGGCAGTGATTCTGTGCGAGCTTTGGCGGTGGACTGCGCCACCGGTGAAGAGATCGCCACCAGC<br>GTAGAGTGGTATCCCCGTTGGCAGAACGGGCAATTTTGTGATGCCCCGAATAACCAGTTCCGTCATCATCCGCGTGACTACATTGAGTCAATGGAAGCGGCGCTGAAAACTGTGC<br>TTGCAGAGCTTAGCGTCGAACAGCGCGCAGCTGTGGTCGGGATTGGCGTTGACACAACCGGCTCGACGCCCGCACCGATTGACGCCGACGGTAACGTCCTGGCGCTGCGCCCGGA<br>GTTTGCCGAAAACCCGAACGCGATGTTCGTATTGTGGAAAGACCACACCGCGGTTGAAGAAGCGGAAGAGATTACCCGTTTGTGCCACGCGCCGGGCAACGTTGACTACTCCCGC<br>TATATTGGCGGTATTTATTCCAGCGAATGGTTCTGGGCAAAAATCCTGCATATAACTCGCCAGGACAACGCCGTGGCACAATCTGCCGCATCGTGGATTGAGCTGTGCGACTGGG<br>TGCCAGCTCTGCTTTCCGGTACCACCCGCCCGCAGGATATTCGTCGCGGACGTTGCAGCGCCGGGCATAAATCTCTATGGCACGAAAGCTGGGGTGGCCTGCCGCCAGCCAGTTT	Primer binding sequence in **bold**

CTTTGATGAGCTGGACCCGATCCTCAACCGTCATTTACCTTCCCCGCTGTTCACTGAAACCTGGACTGCCGATATTCCGGTGGGCACCTTATGCCCGGAATGGGCGCAGCGTCTCG
GCCTGCCTGAAAGCGTGGTGATTTCCGGCGGCGCGTTTGACTGCCATATGGGCGCAGTTGGCGCAGGCGCACAGCCTAACGCACTGGTAAAAGTTATCGGTACTTCCACCTGCGA
CATTCTGATTGCCGACAAACAGAGCGTTGGCGAGCGGGCAGTGAAAGGTATTTGCGGTCAGGTTGATGGCAGCGTGGTGCCTGGATTTATCGGTCTGGAAGCAGGCCAATCGGCG
TTTGGGGATATCTACGCCTGGTTCGGTCGCGTACTCGGCTGGCCGCTGGAACAGCTTGCCGCCCAGCATCCGGAACTGAAAGAGCAAATCAACGCCAGCCAGAAACAACTGCTTC
CGGCGCTGACCGAAGCATGGGCCAAAAATCCGTCTCTGGATCACCTGCCGGTGGTGCTCGACTGGTTTAACGGCCGCCGCACACCGAACGCTAACCAACGCCTGAAAGGGGTGA
TTACCGATCTGAACCTCGCTACCGACGCTCCGCTGCTGTTCGGCGGTTTGATTGCCGCCACCGCCTTTGGCGCACGCGCAATTATGGAGTGCTTCACCGCTCAGGGGATCGCCGTC
AATAACGTGATGGCACTGGGCGGCATCGCGCGCAAAAACCAGGTCATTATGCAGGCCTGCTGCGACGTGCTGAATCGCCCGCTGCAAATTGTTGCTTCTGACCAATGTTGCGCGC
TCGGTGCGGCGATTTTCGCTGCCGTCGCCGCGAAAGTGCACGCAGACATCCCATCAGCCCAGCAAAAAATGGCCAGTGCGGTAGAGAAAACCCTGCAACCGCGCAACGAACAGG
CACAACGCTTTGAACAGCTTTATCGCCGCTATCAGCAATGGGCGATGAGCGCCGAACAACACTATCTTCCAACTTCCGCCCCGGCACAGGCTGACCAGGCCGTTCCGACTCTATA
AGGACACGATAATGACGATTTTTGATAATTATGAAGTGTGGTTTGTCATTGGCAGCCAGCATCTTTACGGCCCGGAGACTCTGCGCCAGGTGACGCAACATGCGGAACACGTTGT
TAATGCACTGAATACAGAAGCGAAGTTGCCCTGCAAACTGGTGCTGAAACCGCTGGGCACCACGCCGGATGAAATCACCGCTATTTGCCGCGACGCGAATTACGACGATCGTTGC
GCTGGTCTGGTGGTGTGGCTGCACACCTTCTCCCCGGCCAAAATGTGGATCAACGGCCTGACCATGCTCAACAAACCGTTGCTGCAATTCCACACCCAGTTCAACGCGGCGCTGCC
GTGGGACAGCATCGACATGGACTTTATGAACCTGAACCAGACCGCGCATGGCGGTCGCGAGTTCGGCTTCATCGGCGCGCGTATGCGTCAGCAACATGCCGTTGTTACCGGTCAC
TGGCAGGATAAACAAGCACATGAGCGTATTGGCTCCTGGATGCGTCAGGCGGTCTCTAAACAGGATACCCGTCATCTGAAAGTCTGCCGTTTTGGCGATAACATGCGTGAAGTAG
CGGTCACCGATGGCGATAAAGTTGCCGCACAGATCAAGTTTGGTTTCTCCGTCAATACCTGGGCGGTTGGCGATCTGGTGCAGGTGGTGAACTCCATAAGCGACGGCGATGTTAA
CGCGCTGGTCGATGAGTACGAAAGCTGCTACACCATGACACCTGCCACACAAATCCACGGCGAAAAACGACAGAACGTGCTGGAAGCGGCGCGTATTGAGCTGGGGATGAAGCG
TTTCCTGGAACAAGGTGGCTTCCACGCTTTCACTACTACCTTTGAAGATTTGCACGGCCTGAAACAGCTTCCGGGTCTGGCCGTACAGCGTCTGATGCAGCAGGGCTACGGCTTTG
CGGGCGAAGGCGACTGGAAAACTGCCGCCCTGCTTCGCATCATGAAGGTGATGTCAACCGGTCTGCAGGGCGGCACCTCCTTTATGGAGGACTACACCTATCACTTCGAGAAAGG
TAATGACCTGGTACTCGGCTCCCATATGCTGGAAGTCTGTCCGTCGATCGCTGTGGAAGAGAAACCGATCCTCGACGTTCAGCATCTCGGTATTGGCGGTAAAGACGATCCTGCCC
GCCTGATCTTCAACACTCAAACCGGTCCAGCCATTGTCGCCAGCCTGATTGATCTCGGCGATCGTTACCGTCTGCTGGTTAACTGCATCGACACGGTGAAAACACCGCACTCCCTG
CCGAAACTGCCGGTGGCGAATGCGCTGTGGAAAGCGCAACCGGATCTGCCGAGCGCTTCCGAAGCGTGGATCCTTGCCGGTGGCGCGCACCATACCGTCTTCAGCCATGCGCTGA
ACCTCAACGATATGCGCCAGTTCGCCGAGATGCACGACATTGAAATCACAGTGATTGATAACGATACCCGCCTTCCAGCGTTTAAAGACGCGCTGCGCTGGAACGAAGTGTATTA
CGGATTTCGTCGCTAAGTAGCCGCATCAGGTATGTAACGCCTGATGCGACGCTGACGCGTCTTATCAGGCCTACGCGTTCCGGATTTGTAGGCCGGATAAGCAAAGCGCATCCGG
CATTCACCGCCTGATGCGGCGCTGGCGCGTCTTATCAGGCCTACGCGTTCCGGATTTGTAGGCCGGATAAGCAAAGCGCATCCGGCATTCACCGCCTGATGCGACGCCGACGCGT
CTTATCAGGCCTACACGCTGCGATTTTGTAGGCCGGATAAGCAAAGCGCATCCGGCACGAAGGAGTCAACATGTTAGAAGATCTCAAACGCCAGGTATTAGAAGCCAACCTGGC
GCTGCCAAAACACAACCTGGTCACGCTCACCTGGGGCAATGTCAGCGCCGTTGATCGCGGGCGCGGCGTCCTGGTGATCAAACCTTCCGGCGTCGACTACAGCATCATGACCGCT
GACGATATGGTCGTGGTCAGCATCGAAACCGGTGAAGTGGTTGAAGGTACGAAAAAGCCCTCCTCCGACACGCCAACTCACCGGCTGCTCTATCAGGCATTCCCGTCTATTGGCG
GCATTGTGCACACACACTCGCGCCACGCCACCATCTGGGCGCAGGCGGGCCAGTCGATTCCAGCAGCCGGCACCACCCACGCCGACTATTTCTACGGCACCATTCCCTGCACCCG
CAAAATGACCGACGCAGAAATCAACGGTGAATATGAGTGGGAAACCGGTAACGTCATCGTAGAAACCTTCGAAAAACAGGGTATCAATGCAGCGCAAATGCCCGGCGTGCTGGT
CCATTCTCACGGCCCATTTGCATGGGGAAAAAACGCCGAAGATGCGGTGCATAACGCCATCGTGCTGGAAGAAGTCGCTTATATGGGGATATTCTGCCGTCAGTTAGCGCCGCAG
TTACCGGATATGCAGCAAACGCTGCTGGATAAACACTATCTGCGTAAGCATGGCGCGAAGGCATATTACGGGCAGTAATGACTGTATAAAACCACAGCCAATCAAACGAAACCA
GGCTATAATCAAGCCTGGTTTTTTATTGGATTTTCAGCGTGGCGCAGGCAGGTTTTATCTTAACCCGACACTGGCGGGACACCCCGCAAGGAACCGAAGTTTCCTTTTGGTTAGCG
ACGGACAACGGGCCGTTGCAGGTTACGCTTGCGCCGCAAGAGTCCGTGGCGTTTATTCCCGCCGATCAGGTTCCCCGCGCCCAGCATATTTTGCAGGGTGAGCAAGGCTTTCGCCT
GACGCCGCTGGCGTTAAAGGATTTTCATCGCCAGCCGGTGTATGGCCTTTACTGTCGCGCCCATCGCCAGTTGATGAATTACGAAAAGCGCCTGCGTGAAGCTGGCGTTACCGTCT
ACGAGGCCGATGTGCGCCCGCCAGAACGCTATCTGATGGAGCGGTTTATCACCTCGCCAGTGTG**GGTCGAGGGTGATATGCGC**

80

Supplementary Table 5. *in vitro* gut-chip model parameters.

**Parameter**	**Value**	**Units**	**Fitted?**
Flow - apical (gut compartment)	60	µL/h	N
Flow - basal (blood compartment)	60	µL/h	N
Volume - apical	40	µL	N
Volume - basal	28	µL	N
Apical Phe input concentration	5	mM (µmol/cm^3)	N
Apical bacterial concentrations	1.25	x 10^8 CFU	N
	6.25	x 10^8 CFU	N
	12.5	x 10^8 CFU	N
V_{max} (Phe → TCA): PAL	0.16	µmol/h/10^8 CFU	Y
K_m (Phe → TCA): PAL	0.2	mM (µmol/cm^3)	N
Bacterial retention time τ (tau)	1.5	h	Y
Permeability (P) - Phe	0.02	cm/h	Y
Permeability (P) - TCA	6 x 10^{-4}	cm/h	Y
Chip Surface area (A)	0.172	cm^2	N

Computational Modeling for *In Vitro* to *In Vivo* Extrapolation and Microfluidics System Validation

Here, we outline computational models of both the *in vitro* microfluidics system and the *in vivo* system, with a focus on relevance to the function of Phe-consuming engineered bacterial strains in non-human primates (NHP). The ability of SYNB1618 to consume Phe *in vivo* in mice and NHP has been demonstrated previously (Isabella *et al.*, 2018). SYNB1618 degrades phenylalanine (Phe) via two distinct mechanisms: (1) the conversion of Phe to *trans*-cinnamic acid (TCA) by phenylalanine ammonia lyase (PAL), and (2) the conversion of Phe to phenylpyruvic acid by L-amino acid deaminase (LAAD). The current study uses the closely related strain, SYN5183, which expresses only a single mechanism for Phe metabolism, by expression of the PAL enzyme.

The motivation for developing these computational models is multi-faceted. Our general purpose is to link *in vivo* and *in vitro* data via shared parameters and to show that the chip can accurately predict the *in vivo* behavior of engineered bacterial therapeutics. Further, we consider this analysis a test case for the development of a modeling workflow to optimize predictive capabilities of *in vitro* models by combining them with suitably tailored *in silico* models. Our more specific purpose is to show that the present gut-chip data can successfully predict metabolic activity of engineered, Phe consuming strains of EcN in the context of NHP, with regard to reduction of Phe uptake from the gut lumen into the blood.

We present a computational model focused on the uptake of Phe from the gut into the blood. This model describes the uptake of Phe derived from dietary protein breakdown, crossing the gut wall by diffusion according to a permeability-surface area product *PA* (cm^3/h; estimated from *in vitro* gut-chip studies) and a concentration differential of Phe between gut and blood. Phe consumption by SYN5183 is described using *in vitro* derived metabolic parameters, assuming Michaelis-Menten kinetics. Estimates of engineered strain activity are then compared to NHP data previously published by Isabella *et al.* (2018) to show that *in vitro* parameters, incorporated into a computational representation of *in vivo* physiology, can successfully predict *in vivo* responses and demonstrate the translational potential of gut-chip microfluidics systems.

In vitro Computational Model Design and Assumptions

In vitro systems mimic certain important, but limited, aspects of human biology. However, computational models can be used to embed *in vitro*-derived parameters, such as gut permeability and the metabolic activity rates of engineered strains, into an overall physiological framework. Parameters that cannot be directly measured can be estimated by fitting a model to experimental data sets. As *in vitro* systems become progressively more sophisticated and incorporate increasing anatomical and physiological detail, fewer model parameters will require estimation by purely computational means. Although no current *in vitro* system takes into account all the complexities of mammalian gastrointestinal physiology, our microfluidics gut-chip system has been modified to closely emulate the critical physiological systems under consideration. Specifically, our flow-through model emulates key mechanical properties such as flow rates and shear stress. It also incorporates key microstructural elements, including relevant gut wall epithelial and capillary endothelial cell types. This system is also able to recreate microvillus structure and the production of an intestinal mucus layer.

Due to the complexity of gut-chip *in vitro* models, an interpretive computational model of the chip itself is helpful to extract relevant parameters (Figure 5a). These key parameters include gut-wall permeability P (cm/h), a key indicator of gut health and a mediator of other physiologically relevant processes, such as water flow, motility, and pH (Cremer *et al.*, 2016, 2017). Our models incorporate P by estimation (for both Phe and TCA) from the chip studies, as described below. P is then incorporated into the *in vivo* model as part of the permeability-surface area product PA (cm^3/h), as described in the *In Vivo Model* section below.

The second critical process informed by our *in vitro* system is SYN5183 metabolic activity. The *in vitro* computational model takes into account the kinetics in the chip, both of Phe and SYN5183, under the various dosing scenarios outlined in the main text. Incorporating estimates of P from control (no ECN) study arms and using K_m (mM) values for the PAL pathway from literature (Isabella *et al.*, 2018), we are able to fit a single V_{max} [µmol/(CFU-h)] value to data from various doses of ECN. An important consideration is that SYN5183, used in our gut- chip studies, expresses PAL but not LAAD. However, TCA (and HA), which are produced exclusively via the PAL pathway, can be used to assess the relevance and validity of the *in vitro* studies.

Three *in vitro* SYN5183 and Phe dosing designs, described in detail in the main text, were utilized for gut-chip studies. Briefly, they are (1) continuous infusion of both the Phe and SYN5183 into the (apical) gut compartment; (2) bolus dosing of SYN5183 via the apical route, followed by infusion of Phe into the apical compartment; and (3) bolus dosing of SYN5183 via the apical route, followed by infusion of Phe into the basolateral (blood) compartment. Scenarios (2) and (3) were designed to emulate the elimination of Phe by SYN5183 directly from the gut (food) and indirectly from the blood, respectively. As described in the main text, flow rates in both compartments were set to 60 µL/h. The retention (half-life) of SYN5183 seeded into the gut compartment was found to be less than 3 hours, and was taken to be approximately 1.5 h at this flow rate (see Figure 2c of the main text). Phe was infused (5 mM) in gut (apical compartment) to simulate a meal (scenario 2), or into the blood (basolateral) compartment at 1 mM (scenario 3).

In Vitro Model Equations

In the apical (gut) compartment (volume V_G), time-courses of Phe and TCA concentrations (C^G_{Phe}, C^G_{TCA} respectively) are represented by Equations S1 and S2, respectively.

$$V^G \frac{dC^G_{Phe}}{dt} = F_G . C^G_{Phe,i} - \frac{V_{max} . C_{bact} . C^G_{Phe}}{C^G_{Phe} + K_m} - PA_{Phe} . \left(C^G_{Phe} - C^B_{Phe}\right) - F_G . C^G_{Phe}$$

Equation S1

$$V^G \frac{dC^G_{TCA}}{dt} = \frac{V_{max} . C_{bact} . C^G_{Phe}}{C^G_{Phe} + K_m} - PA_{TCA} . \left(C^G_{TCA} - C^B_{TCA}\right) - F_G . C^G_{TCA}$$

Equation S2

where F_G is the flow through the gut compartment, PA is the permeability-surface area product of the barrier separating the compartments, and V_{max} and K_m are Michaelis-Menten parameters for the conversion of Phe

to TCA by SYN5183. Parameters for the *in vitro* model, including dimensions, flows, and volumes, as well as the fitted parameters P and V_{max} are given in Supplementary Table 5.

Note that the total metabolic capacity for converting Phe to TCA is given by the product of the bacterial concentration in the gut, C_{bact} (in units, for example, of 10^8 CFUs) and the V_{max} per bacterial unit (10^8 CFUs). C_{bact} is determined by the infusion method; we use both a steady infusion of SYN5183 and a bolus dosing technique in which bacteria are established in the gut compartment and are then slowly washed out from the system (see main text). We assume this washout follows a simple exponential profile, with a characteristic residence time τ, according to the following equation:

$$C_{bact} = C_{bact,i}e^{-t/\tau} \qquad \text{Equation S3}$$

Initial bacterial concentrations $C_{bact,i}$ are 1.25, 6.25, and 12.5 (x 10^8 CFU/ml) for the low, medium, and high doses respectively, and τ (*tau* in the code) is the residence time of bacteria in the chip. This latter seeding strategy more closely emulates the physiological situation *in vivo*. Sample model code is provided for both probiotic dosing scenarios.

Equations S4 and S5 describe the kinetics of Phe and TCA (concentrations $C^B{}_{Phe}$ and $C^B{}_{TCA}$) in the basolateral (blood) compartment (volume V_B, flow F_B):

$$V^B \frac{dC^B_{Phe}}{dt} = PA_{Phe}.\left(C^G_{Phe} - C^B_{Phe}\right) - F_B.C^B_{Phe} \qquad \text{Equation S4}$$

$$V^B \frac{dC^B_{TCA}}{dt} = PA_{TCA}.\left(C^G_{TCA} - C^B_{TCA}\right) - F_B.C^B_{TCA} \qquad \text{Equation S5}$$

In Vitro Model Results

Representative simulations of the output concentrations of Phe and TCA from the chip, compared with experimental data, are shown in Supplementary Figure 4. Briefly, an estimate of P from gut to blood for Phe of 1.5 x 10^{-7} cm/s ~ 5 x 10^{-4} cm/h was estimated from the *in vitro* chip studies (Figure 3). The surface area A and transit time τ were independently fitted to the control (CFU = 0) time-course data, giving estimates of A~630cm^2 and τ ~ 1.4 h. Metabolic parameters for Phe metabolism by SYN5183 from the chip studies, at all dose levels (1.25 x 10^8, 6.25 x 10^8, and 1.25 x 10^9 CFUs), yielded an estimate of V_{max} for the SYN5183 construct of 0.16 umol/10^8 CFU/h. K_m for the PAL pathway was taken as 0.2 mM, obtained from previous *in vitro* assays.

In Vivo Model and Assumptions

The *in vivo* model was derived to describe SYN5183 (or SYNB1618) administration in NHP and compared to data from Isabella *et al.* (2018). Isabella *et al.* (2018) used a 5.0 g peptone bolus to deliver Phe to fasted NHP. Assuming 5% peptide bound Phe, this results in the administration of 0.25 g (1.5 millimole) Phe. This Phe bolus was given alone and in conjunction with one of 3 doses of SYNB1618: 1.8 x 10^{11}, 3.6 x 10^{11}, or 7.2 x 10^{11} CFU. Figure 5b shows a schematic of our *in vivo* model describing the uptake of Phe

from the gut into the blood, together with the impact of SYNB1618. As the food bolus moves through the gut, Phe is absorbed in the blood according to a gut wall permeability-surface area product, *PA* (cm^3/h). If SYNB1618 is present, Phe is also metabolized to TCA. After transit time τ (h), the bolus exits the gut.

In Vivo Model equations

Considering the case in which Phe is administered into the gut when no SYNB1618 is present. The rate of change of Phe in blood (serum) c_b (mM or µmol/cm^3) is given by Equation S6,

$$V_b \frac{dc_b}{dt} = PA(c_g - c_b) + D - kc_b \qquad \text{Equation S6}$$

where V_b is the blood volume (cm^3), D is rate of Phe accumulation due to tissue protein breakdown (µmol/h), and PA (cm^3/h) is the permeability-surface area product. k (cm3/h) represents the removal rate (clearance) of Phe from the blood by the liver and kidney by first-order kinetics.

In the fasting steady-state (SS), dc_b/dt=0, and assuming that there is negligible input of Phe from the gut in the fasting state, then $k = D/c_{b,ss}$, where $c_{b,ss}$ is the fasting SS blood level. From Isabella *et al.* (2018), Figure 6D, this blood serum level is reached at late times after bolus infusion and is approximately 0.05 mM. From Kaufman (1999), D = 70umol/kg/12h ~ 36 umol/h (estimated in humans). Note that we assume that input of Phe into the blood in SS is primarily due to protein breakdown, D, and that influx of Phe from the gut to the blood in the (fasting) SS is not significant by comparison.

Thus, substituting for k, Equation S6 becomes:

$$V_b \frac{dc_b}{dt} = PA(c_g - c_b) + D(1 - \frac{c_b}{c_{b,ss}}) \qquad \text{Equation S7}$$

Similarly, for Phe in the gut (c_g), we represent the rate of change of concentration by the outflux across the gut wall:

$$V_g \frac{dc_g}{dt} = -PA(c_g - c_b) \qquad \text{Equation S8}$$

Here, V_g is the volume of bolus (not total gut volume), which is assumed spread out as it passes through the gut. Although the volume of the bolus as administered is available, because of this dispersion and the presence of gastric and pancreatic secretions, we fit V_g to the data. In general, V_g will be somewhere between the administered bolus volume and that of the gut volume itself. Note that the effective surface area A for absorption is correlated with V_g. For example, $A \sim 2\ V_g / r$ for a cylindrical gut, radius r. As the bolus disperses, A increases, but is compensated by Phe concentration decreases, since flux is proportional to the product of concentration times area. When the bolus of Phe is followed by a bolus of engineered cells, we need to consider the degree of "overlap" between them in order to reflect the strain's access to Phe. Finally, the bolus of Phe has a finite transit time through gut, designated by τ, which is also considered a fitted parameter.

Now we turn to the reduction of Phe uptake from the gut to the blood by SYNB1628 metabolic activity. Equation S8 is replaced with the following:

$$V_g \frac{dc_g}{dt} = -PA(c_g - c_b) - c_{SYNB1618}\left(\frac{V_{max,PAL} \cdot c_g}{c_g + K_{m,PAL}}\right) - c_{SYNB1618}\left(\frac{V_{max,LAAD} \cdot c_g}{c_g + K_{m,LAAD}}\right) \qquad \text{Equation S9}$$

Here $C_{SYNB1618}$ is the concentration of SYNB1618 in the administered bolus, and V_{max} and K_m are the Michaelis-Menten parameters for the PAL and LAAD pathways expressed by the engineered strain.

In Vivo Model Results

We employ the following strategy for the implementation of the *in vivo* Phe model. First, we develop a simple model of blood Phe levels following bolus administration without SYNB1618 administration (control), but with normal liver metabolism to establish the background kinetics of Phe. We then look at the impact, in terms of modified blood Phe concentrations, due to the introduction of SYNB1618 in the gut, using *in vitro* metabolic parameters, while holding other parameter values constant. The predicted impact of SYNB1618 administration to blood Phe concentrations can then be compared with experimental data from NHP studies (Isabella et al (2018)).

Simulations from our *in vivo* model are compared with the NHP data of Isabella *et al.* (2018) in Figure 5 of the main text**. Figure 5c-d** show non-human primate (NHP) blood (serum) data and model simulations, assuming PAL enzyme activity only for SYNB1618 (V_{max} = 0.16 μmol/10^8 CFU/h, K_m = 0.2 mM). A 5g peptone bolus administered to fasting NHPs, together with a dose 0 (control), 1.8 x 10^{11}, 3.6 x 10^{11}, and 7.2 x 10^{11} CFU SYNB1618. Experimental data are represented by points with error bars. Solid lines display the corresponding *in vivo* model simulations. Fitted parameters are the effective gut wall surface area *A* (20 cm^2) and the transit time τ (1.4 h). Note that 20 cm^2 seems to be a reasonable estimate for the effective gut wall surface area of a 10 ml bolus, corresponding to an effective cylinder in the small intestine of 1 cm radius and length about 3.2 cm. These control values for P, A, and τ were retained at the other non-zero doses of SYNB1618 in NHP.

The slight underestimation of Phe uptake predicted by these initial simulations (Figure 5c-d) was particularly pronounced at the lower ECN concentrations, and may be due to the absence in the model of the second enzyme, LAAD (present in the SYNB1618 construct but absent in the SYN5183 construct used in the chip studies). Figure 5e shows the impact of adding LAAD enzyme activity [V_{max} = 0.32 μmol/10^8 CFU/h, fitted to the data manually, using K_m = 2.3 mM (Pantaleone *et al.*, 2001)] to the model, improving the overall fit. Figure 5e-f show comparisons of predicted versus measured reductions of blood Phe in NHP from the *in vivo* computational model, with and without the LAAD enzyme in the model. Adding the second enzyme improves the fit, with the Pearson correlation coefficient, R^2, increasing from 0.55 to 0.70)

References:

1. Cremer, J., Segota, I., Yang, C.-Y., Arnoldini, M., Sauls, J. T., Zhang, Z., Gutierrez, E., *et al.* (2016). Effect of flow and peristaltic mixing on bacterial growth in a gut-like channel. Proceedings of the National Academy of Sciences of the United States of America, 113(41), 11414–11419. doi:10.1126/science.112.2920.715

2. Cremer, J., Arnoldini, M., & Hwa, T. (2017). Effect of water flow and chemical environment on microbiota growth and composition in the human colon. Proceedings of the National Academy of Sciences of the United States of America, 114(25), 6438–6443. doi:10.1021/mp100198q

3. Isabella, V. M., Ha, B. N., Castillo, M. J., Lubkowicz, D. J., Rowe, S. E., Millet, Y. A., Anderson, C. L., *et al.* (2018). Development of a synthetic live bacterial therapeutic for the human metabolic disease phenylketonuria. Nature biotechnology, 1–17. Nature Publishing Group. doi:10.1038/nbt.4222

4. Kaufman, S. (1999). A model of human phenylalanine metabolism in normal subjects and in phenylketonuric patients. Proceedings of the National Academy of Sciences of the United States of America, 96, 3160–3164.

5. Pantaleone, D. P., Geller, A. M., and Taylor, P. P. (2001) Purification and characterization of an L-amino acid deaminase used to prepare unnatural amino acids. J. Mol. Catal. B: Enzym. 11, 795–803
