# Supplementary material for: Characterization of an engineered live bacterial therapeutic for the treatment of phenylketonuria in a human gut-on-a-chip
Source: Nat Commun. 2021 May 14;12:2805. doi: 10.1038/s41467-021-23072-5 (PMC8121789; doi:10.1038/s41467-021-23072-5)
Supplement: Supplementary file 2 — Reporting Summary [file 41467_2021_23072_MOESM2_ESM.pdf]

nature research

Corresponding author(s): Mark Tyler Nelson

Last updated by author(s): 04/08/2021

Reporting Summary

Nature Research wishes to improve the reproducibility of the work that we publish. This form provides structure for consistency and transparency in reporting. For further information on Nature Research policies, see our Editorial Policies and the Editorial Policy Checklist.

Statistics

For all statistical analyses, confirm that the following items are present in the figure legend, table legend, main text, or Methods section.

n/a	Confirmed	
☐	☒	The exact sample size (n) for each experimental group/condition, given as a discrete number and unit of measurement
☐	☒	A statement on whether measurements were taken from distinct samples or whether the same sample was measured repeatedly
☐	☒	The statistical test(s) used AND whether they are one- or two-sided<br>*Only common tests should be described solely by name; describe more complex techniques in the Methods section.*
☒	☐	A description of all covariates tested
☒	☐	A description of any assumptions or corrections, such as tests of normality and adjustment for multiple comparisons
☒	☐	A full description of the statistical parameters including central tendency (e.g. means) or other basic estimates (e.g. regression coefficient) AND variation (e.g. standard deviation) or associated estimates of uncertainty (e.g. confidence intervals)
☐	☒	For null hypothesis testing, the test statistic (e.g. F, t, r) with confidence intervals, effect sizes, degrees of freedom and P value noted<br>*Give P values as exact values whenever suitable.*
☒	☐	For Bayesian analysis, information on the choice of priors and Markov chain Monte Carlo settings
☒	☐	For hierarchical and complex designs, identification of the appropriate level for tests and full reporting of outcomes
☐	☒	Estimates of effect sizes (e.g. Cohen's d, Pearson's r), indicating how they were calculated

Our web collection on statistics for biologists contains articles on many of the points above.

Software and code

Policy information about availability of computer code

Data collection	- PRISM v8, GraphPad, statistical analysis and graphing software was used to analyze results and construct figures<br>- Microsoft office (Word, Excel) were used for generating the manuscript and organizing RAW results<br>- Endnote X9 Mac was used to format references and produce a bibliography<br>- Adobe Illustrator v8 used to format and organize figures<br>- Nikon Elements (NIS ElementsAR ver. 4.6.0.) Nikon NIS- Elements Viewer Basic Research acquisition software; 3D deconvolution application package<br>- ImageJ, image analysis software, open-source v1.51<br><br>In vitro to In vivo extrapolation: Computational models were custom designed to characterize the activity of the microbial strain in a human gut-chip system and extrapolate those results to prior published data. Ordinary differential equations were solved using open source, Berkeley Madonna 10.0 c-based software.
Data analysis	- In vitro to In vivo extrapolation: Gut-chip permeability, gut to blood phe transport, SYN51S3 activity and metabolism in the gut chip, and the extrapolation of gut-chip results to published non-human primate results were analyzed in Berkeley Madonna 10.0, were Pearson's correlation coefficient was also statistically produced.<br><br>- PRISM vs statistical analysis and graphing software was utilized to process and present all other results

For manuscripts utilizing custom algorithms or software that are central to the research but not yet described in published literature, software must be made available to editors and reviewers. We strongly encourage code deposition in a community repository (e.g. GitHub). See the Nature Research guidelines for submitting code & software for further information.

Data

Policy information about availability of data

All manuscripts must include a data availability statement. This statement should provide the following information, where applicable:

- Accession codes, unique identifiers, or web links for publicly available datasets
- A list of figures that have associated raw data
- A description of any restrictions on data availability

The datasets and associated source data files generated during and/or analyzed during the current study are available from the corresponding author (mark.nelson.35@us.af.mil) on reasonable request.

Field-specific reporting

Please select the one below that is the best fit for your research. If you are not sure, read the appropriate sections before making your selection.

[x] Life sciences [ ] Behavioural & social sciences [ ] Ecological, evolutionary & environmental sciences

For a reference copy of the document with all sections, see nature.com/documents/nr-reporting-summary-flat.pdf

Life sciences study design

All studies must disclose on these points even when the disclosure is negative.

Sample size	For experiments involving quantification of macrovillus structures, analysis of gut or blood effluents, and numbers of viable cells in the gut or endothelial compartments, n=3 was chosen as the minimal replicate number, and sample size was determined by the number regions of interests we measured within a macrovillus image. We determined this to be sufficient owing to internal control and low observed variability between samples.
Data exclusions	Data were not excluded from analysis.
Replication	All replication attempts were found to be successful, 9 gut chips per condition were built and all chips built presented homogeneous macrovillus structures, stained positively for tight junction markers, and displayed tight standard deviations for analytical tests.
Randomization	48 gut chips were built at once, chips were selected at random and assigned an experimental condition; however all cells and effluents were analyzed evenly without sub-sampling and thus randomization was not applied.
Blinding	Blinding for sample collection and analysis was not possible due to image analysis; however, all samples were analyzed evenly and without sub-sampling. All LC-MS/MS samples were blinded and characterized as such.

Reporting for specific materials, systems and methods

We require information from authors about some types of materials, experimental systems and methods used in many studies. Here, indicate whether each material, system or method listed is relevant to your study. If you are not sure if a list item applies to your research, read the appropriate section before selecting a response.

Materials & experimental systems

n/a	Involved in the study	
[ ]	[x]	Antibodies
[ ]	[x]	Eukaryotic cell lines
[x]	[ ]	Palaeontology and archaeology
[x]	[ ]	Animals and other organisms
[x]	[ ]	Human research participants
[x]	[ ]	Clinical data
[x]	[ ]	Dual use research of concern

Methods

n/a	Involved in the study	
[x]	[ ]	ChIP-seq
[x]	[ ]	Flow cytometry
[x]	[ ]	MRI-based neuroimaging

Antibodies

Antibodies used	Zona Occludin-1 (ZO-1), Invitrogen, 33-9100, 5 ug/mL<br>Secondary Goat anti-Mouse IgG (H+L), Superclonal Recombinant Antibody, Alex Fluor 488, Invitrogen, A28175, 5 ug/mL<br>Secondary Goat anti-Mouse IgG (H+L), Superclonal Recombinant Antibody, Alex Fluor 555, Invitrogen, A28180, 5 ug/mL<br>25-plex Human ProcartaPlex(TM) Panel 1B, EPX250-12166-901, Thermo Fisher Scientific USA: GM-CSF; IFN alpha; IFN gamma; IL-1 alpha; IL-1 beta; IL-1RA; IL-2; IL-4; IL-5; IL-6; IL-7; IL-9; IL-10; IL-12 p70; IL-13; IL-15; IL-17A; IL-18; IL-21; IL-22; IL-23; IL-27; IL-31; TNFalpha; TNF beta/LT, antibodies were added to the assay using manufacturers suggested dilution: 1 to 10

Validation	ZO-1 (Invitrogen, Cat. #33-9100): was validated by the manufacturer, Invitrogen, by Knockdown to ensure that the antibody binds to the antigen stated. Antibody specificity was demonstrated by siRNA mediated knockdown of target protein. Caco-2 cells were transfected with ZO-1 siRNA and decrease in signal intensity was observed in ICC application using Anti-ZO-1 Monoclonal Antibody (ZO1-1A12), as stated by the manufacturer.<br><br>25-plex Planel 1B ProcartaPlex Cytokine ELISA (ThermoFisher Scientific, Cat.# EPX250-12166-901): was validated by the manufacturer, FisherScientific showing a high degree of specificity and species dependent analyte binding, in addition each lot of standards and antibodies were rigoursly quality controlled, and were provided with a certificate of analysis indicating the validation of the assay. In addition, we utilized a dual standard curve in each plate, resulting in an R2 value greater than or equal to 99.5%. The corresponding experimental results also displayed values that were within the standard curve and complied with assay absolute limits.

Eukaryotic cell lines

Policy information about cell lines

Cell line source(s)	Caco-2 ATCC HTB-37<br>HT29-MTX-E12 Cells, Millipore-Sigma, 12040401<br>Human Microvascular endothelial cells - hTert immortalized, ATCC TIME hTERT, CRL-4045
Authentication	Authentication was performed by the vendors, providing a full karyotyping and STR profile of each cell line provided, authenticating the source and validity of the cell line.
Mycoplasma contamination	All cell lines were mycoplasma free as determined by PCR method and tested weekly for continued surveillance
Commonly misidentified lines<br>(See ICLAC register)	No commonly misidentified lines were used in this study.
